# A Holistic Approach to Human-Supervised Humanoid Robot Operations in Extreme Environments

**DOI:** 10.3389/frobt.2021.550644

**Published:** 2021-06-18

**Authors:** Murphy Wonsick, Philip Long, Aykut Özgün Önol, Maozhen Wang, Taşkın Padır

**Affiliations:** ^1^ Institute for Experiential Robotics, Northeastern University, Boston, MA, United States; ^2^ Irish Manufacturing Research, National Science Park, Mullingar, Ireland

**Keywords:** humanoid robots, motion planning, supervised autonomy, nuclear, glovebox

## Abstract

Nuclear energy will play a critical role in meeting clean energy targets worldwide. However, nuclear environments are dangerous for humans to operate in due to the presence of highly radioactive materials. Robots can help address this issue by allowing remote access to nuclear and other highly hazardous facilities under human supervision to perform inspection and maintenance tasks during normal operations, help with clean-up missions, and aid in decommissioning. This paper presents our research to help realize humanoid robots in supervisory roles in nuclear environments. Our research focuses on National Aeronautics and Space Administration (NASA’s) humanoid robot, Valkyrie, in the areas of constrained manipulation and motion planning, increasing stability using support contact, dynamic non-prehensile manipulation, locomotion on deformable terrains, and human-in-the-loop control interfaces.

## 1 Introduction

As the worldwide energy demand is expected to increase by 50% within the next 3 decades, nuclear energy will play a critical role in meeting clean energy targets worldwide. At the same time, many of the world’s nuclear reactors are aging; from Japan to the United Kingdom to the United States, scientists, engineers and regulators are counting on new innovative technologies that will make decommissioning and clean-up missions safe for humans, environmentally-friendly and cost-effective. Furthermore, industrializing countries are investing in building new nuclear power plants to meet their growing energy demands.

Nuclear energy operations and nuclear disasters have great international impact with no boundaries. Ensuring safe, efficient, and productive operations of facilities and improving response to unplanned emergencies at any location around the globe is in the best interest of the international community. Moreover, the urgency and scale of the problems identified in high-consequence situations, such as the Fukushima (Japan) clean up and waste tank decommissioning in Savannah River Site (United States), require an interdisciplinary team of scientists, engineers, and technologists to solve similar yet sufficiently distinct challenges. For example, since 1989, the United States Department of Energy has spent over *$*250 billion of public funds on cleanup. The cleanup is less than half complete and the remaining mission scope is estimated to cost at least another *$*250 billion more over a 40–50- year period. Similarly, the Japanese government estimated the total costs for the Fukushima cleanup at *$*188 billion for the next 40 years.

It is now clear that robotics will play a key role in accelerating these cleanup timelines and reducing the costs, by addressing operational needs and challenges in nuclear facilities. Robotics technologies are needed to remotely access nuclear and other highly hazardous facilities under human supervision to perform inspection and maintenance tasks during normal operations. During decommissioning, robots will become the eyes and hands of human operators in places no human has gone for more than 50 years. However, using robots in such environments is not without challenges, the Fukushima disaster has shown that conventional robots must be significantly modified, Nagatani et al. (2011b), to cope with highly radioactive environments. This is typically achieved by equipping the system with lead plates to protect electrical components, Nagatani et al. (2011a), which in-turn add weight and may impair functionality. Alternatively, the number of electronic components can be minimized and used in conjunction with specifically hardened parts, however high levels of radiation still can destroy such systems in a matter of days, Funakoshi (2016), Urabe and Stapczynski (2017), Yirmibeşoğlu et al. (2019). Nevertheless, robots have already been deployed in less active nuclear environments in commissioning and waste disposal task, Bogue (2011), and on-going research is demonstrating promising applications for human-supervised robotics in a range of different tasks in the future, Marturi et al. (2016). They will improve our ability to respond to and recover from unplanned events or operational emergencies in such critical and safety-significant applications. Nuclear environments are *dangerous* for humans to operate in due to the presence of highly radioactive materials. They are typically *distant* as the facilities separate these dangerous environments from humans by thick walls. And they present *daring* operational conditions by size and configuration with tight passages, debris accumulated over the years and cluttered internals.

Our research plan is motivated by the need for general purpose robots in routine and emergency operations in nuclear facilities for: 1) Disaster response and environmental clean-up, such as more than 175 waste tanks in Hanford (WA) or the F-canyon in Savannah River (SC). These processes rely heavily on accurate remote sampling and characterization before permanently grouting the facilities, by collecting samples from different spots for analyzing what and how much radioactive material remains. The operating environment is unknown and cluttered with vertical and horizontal piping, fallen debris and puddles and muck-like material on the floors (in the waste tanks). 2) Operational efficiencies—federal laws require nuclear facilities to develop and maintain emergency preparedness plans to protect the public. These emergencies include unusual events during normal operations to black swan events such as Fukushima, Chernobyl and Three Mile Island accidents. Furthermore, the aging workforce in the energy sector requires the adoption of technology to keep up with day-to-day operations. As a result, human-supervised robot assets with robust manipulation capabilities in these challenging environments and situations are needed. 3) Worker safety—Before any work can begin, human workers must enter a facility to characterize radioactive hazards, such as type of radiation, dose rates, and location of sources. This data is then used to determine the proper protective clothing and stay time limits for personnel. Replacing personnel with robots would be highly desirable in radioactive environments.

This paper discusses our approach to move towards utilizing humanoid robots in nuclear energy operations and nuclear disasters by furthering the development of human-supervised robot control and manipulation capabilities. Section 2 presents a method capable of handling manipulation and motion planning in constrained environments, such as gloveboxes. Section 3 presents work in utilizing support contacts to increase stability of a standing humanoid robot operating inside a glovebox. Section 4 presents a method to plan dynamic non-prehensile manipulation behaviors in a highly-constrained environment, with focus on gloveboxes. Section 5 presents a way to estimate *in-situ* deformable terrains in order to navigate in unknown environments. Section 6 presents a human-in-the-loop user interface for operating humanoid robots.

## 2 Constrained Manipulation and Motion Planning

In hazardous environments it is crucial to perform manipulation tasks effectively. Additionally, such environments often provide constraints on the motions allowed, such as when operating through glovebox ports. In order to accomplish effective manipulation, different robot configurations should be evaluated. This is particularly important for redundant systems such as humanoid robots. A robot performance measure can be classified as local, e.g., manipulability (Yoshikawa, 1984) or global such as workspace analysis (Vahrenkamp and Asfour, 2015). Local indexes are advantageous as they provide a more generic solution and may be utilized in control frameworks without workspace knowledge. Hence, they can be used to choose a configuration based on a robot’s inherent capability. However, local indexes study the system’s kinematic transformations from configuration to task space, ignoring environmental constraints that have significant effects on the robot’s admissible motions. In particular, for operations in hazardous environments, the workspace is often unstructured and uncontrolled. Moreover, unscheduled contacts may lead to catastrophic results. For these reasons, it is important to transmit an accurate measure of what the robot can or cannot do in its current pose to a remote operator/supervisor.

The following section recalls the work presented in (Long and Padır, 2018; Long and Padir, 2019; Long et al., 2019) in which a new measure called the constrained manipulability polytope (CMP) that considers the system’s kinematic structure, including closed chains, composite sub-mechanisms, joint limits, and the presence of obstacles is developed.

### 2.1 Related Work

The manipulability ellipsoid first defined (Yoshikawa, 1984) measures the capabilities of a robot manipulator based on its kinematic structure. Extensions to include positional joint limits are proposed in (Tsai, 1986) using penalty functions and (Abdel-Malek et al., 2004) using augmented jacobian matrices. Robots with heterogeneous joint velocity limits are examined in (Lee, 1997), while dynamic constraints are considered (Bowling and Khatib, 2005; Zollo et al., 2008). For humanoid robots, improvements on local measures can be obtained by including the effects of contact while evaluating the dynamic manipulability of a humanoid’s center of mass (Gu et al., 2015; Azad et al., 2017). Moreover in (Azad et al., 2017) either joint torque and/or acceleration limits are accounted for by using a scaling matrix. In (Vahrenkamp et al., 2012, 2013) the manipulability ellipsoid is augmented to include environmental constraints. The authors include joint position limits and the detrimental effects of nearby obstacles using a spatial decomposition referred to as the hyperoctants approach. Manipulability polytopes (Kokkinis and Paden, 1989) provide a more elegant and indeed exact method for representing velocity limits in the Cartesian space. There are several examples where diverse constraints, defined by a set of inequality or equality equations, have been incorporated into polytopes. For instance, mobile robot toppling constraints have been integrated into the available wrench set for a cable-driven parallel robot in (Rasheed et al., 2018). Alternatively, friction constraints can be added after linearization (Caron et al., 2017).

### 2.2 Manipulability

Consider an *n* degree-of-freedom (DOF) manipulator in *m* dimensional space. Let $\nu_{n}$ denote the twist at the end effector, comprising three translational and three angular velocities defined, respectively, as v and ω. $\nu_{n}$ is obtained as

$$\nu_{n}=\left[\begin{array}{c} v\\ \omega \end{array}\right]=J_{n}\dot{q},$$ (1)

where $J_{n}\in {\mathbb{R}}^{6\times n}$ is the Jacobian matrix and $\dot{q}={\left[{\dot{q}}_{1},{\dot{q}}_{2}\ldots {\dot{q}}_{n}\right]}^{T}$ is the joint velocity vector. An exact measure of the manipulator’s capabilities can be obtained by studying the manipulability polytope in conjunction with the joint velocity limits. A polytope, P can be represented as the convex hull of its vertex set (V-representation), i.e.,

$$P^{V}=\left\{x:x=\overset{n}{\underset{i=1}{\sum }}\alpha_{i}y_{i}|\alpha_{i}\geq 0,\overset{n}{\underset{i=1}{\sum }}\alpha_{i}=1\right\},$$ (2)

where $y_{i}$ denotes the $i^{th}$ element of the vertex set and x is any point inside P. Equivalently, P can be defined as the volume bounded by a finite number of half-spaces (ℋ-representation)

$$P^{H}=\mathrm{Ax}\leq b,$$ (3)

where A contains the half-spaces’ normals and b is the shifted distance from the origin along the normal. Converting from V and ℋ is possible, for example, using the double description^1^ method (Fukuda and Prodon, 1996). The polytope representing joint velocities for an *n*-DOF robot, denoted by Q, is written in ℋ-representation as

$$Q^{H}=\left[\begin{array}{c} I_{n}\\ -I_{n} \end{array}\right]\dot{q}\leq \left[\begin{array}{c} {\dot{q}}_{max}\\ -{\dot{q}}_{min} \end{array}\right],$$ (4)

where $I_{n}$ is the n×n identity matrix and ${\dot{q}}_{max}$ and ${\dot{q}}_{min}$ denote the robot’s maximum and minimum joint velocities respectively. The equivalent polytope defined by its vertices is written as

$$Q^{V}=\left\{\begin{array}{ccc} {\dot{q}}_{1}^{v}, & {\dot{q}}_{2}^{v}, & \ldots ,{\dot{q}}_{2^{n}}^{v} \end{array}\right\},$$ (5)

where ${\dot{q}}_{i}^{v}$ denotes the $i^{th}$ vertex of Q. The convexity of a polytope is preserved under affine transformation, i.e., a linear transformation applied to $Q^{V}$ is a convex combination of the same linear transformation applied to the vertices. Thus, a manipulability polytope (MP), denoted as P, representing the Cartesian-space velocities can be obtained using the linear kinematic transform defined by one. P’s vertex set representation is given as

$$P^{V}=\left\{\begin{array}{cc} \nu_{1}^{v} & \ldots \nu_{2^{n}}^{v} \end{array}\right\}=\left\{\begin{array}{cc} J_{n}{\dot{q}}_{1}^{v}\ldots & J_{n}{\dot{q}}_{2^{n}}^{v} \end{array}\right\}$$ (6)

and its volume, denoted as $w_{p}$, can be used as an indicator of robot performance.

### 2.3 Constrained Manipulability

The manipulability polytope does not give a true picture of the robot’s capabilities as they may be reduced due to environmental or joint limit constraints. Thus in our previous work (Long and Padır, 2018; Long and Padir, 2019), a method of considering obstacle and joint position limits is given. To do so the *kineostatic danger field* (Ragaglia et al., 2014) as an input which limits the maximum attainable velocity in the direction of a potential collision. The robot’s velocity is reduced until the danger-field value is below a predefined threshold. The *kineostatic danger field* divides the robot’s links into *l* control points (CPs) and the workspace into *c* cells. The danger field for the $j^{th}$ (j=1,…,c) cell is calculated as

$$\phi_{j}=\underset{i=1\ldots l}{\text{max}}\left(\frac{1}{\left\|r_{i}-r_{j}\right\|}+\frac{\left|\left|v_{i}\right|\right|\text{cos}\left(∠\left(r_{i}-r_{j},v_{i}\right)\right)}{{\left|\left|r_{i}-r_{j}\right|\right|}^{2}}\right),$$ (7)

where $r_{j}$ and $r_{i}$ denote the position vector of the $j^{th}$ cell and the robot’s $i^{th}$ CP, respectively. The translational velocity of point *i* is denoted by $v_{i}$. To generate a set of inequality constraints, Eq. 7 is re-defined as

$$\forall i\in \text{CP},\phi_{j}\leq \frac{1}{\left|\left|r_{ij}\right|\right|}+\frac{\left|\left|v_{i}\right|\right|\text{cos}\left(∠\left(r_{ij},v_{i}\right)\right)}{{\left\|r_{ij}\right\|}^{2}},$$ (8)

where $r_{ij}=r_{i}-r_{j}$. Substituting the dot product

$$\text{cos}\left(∠\left(r_{ij},v_{i}\right)\right)=\frac{v_{i}^{T}r_{ij}}{\left|\left|v_{i}\right|\right|\left|\left|r_{ij}\right|\right|},$$ (9)

(8) becomes

$$\phi_{j}\leq \frac{1}{\left|\left|r_{ij}\right|\right|}+\frac{v_{i}^{T}r_{ij}}{{\left|\left|r_{ij}\right|\right|}^{3}},$$ (10)

Finally, by introducing ${\hat{r}}_{ij}$ the normalized unit vector of $r_{ij}$, Eq. 10 becomes

$$v_{i}^{T}{\hat{r}}_{ij}\leq \phi_{j}{\left|\left|r_{ij}\right|\right|}^{2}-\left|\left|r_{ij}\right|\right|$$ (11)

The robot’s velocity is reduced until the danger field value at the obstacle location, denoted as *o* is below a threshold, i.e., a desired danger value. Eq. 11 is re-written as

$$v_{i}^{T}{\hat{r}}_{io}\;\;\leq \phi^{d}{\left|\left|r_{io}\right|\right|}^{2}-\left|\left|r_{io}\right|\right|,$$ (12)

$r_{o}$ is the obstacle’s position vector with respect to the robot’s fixed frame and $\phi^{d}$ denotes desired danger value. By introducing Eq. 1, the following expression is obtained in configuration space

$${\hat{r}}_{io}^{T}\;J_{i}\dot{q}\;\;\;\leq \phi^{d}{\left\|r_{io}\right\|}^{2}-\left\|r_{io}\right\|,$$ (13)

where $J_{i}\in {\mathbb{R}}^{3\times n}$ is the Jacobian matrix at point *i*. The Jacobian matrix at any CP will have *n* columns. However, if a joint does not contribute to the velocity at the $i^{th}$ CP, the $i^{th}$ column of contains only zeros. Hence, Eq. 13 constrains the maximum velocity for the $i^{th}$ CP in the direction toward the obstacle. Taking into account the *l* CPs leads to the following set of inequalities

$$\left[\begin{array}{c} {\hat{r}}_{1o}^{T}\;J_{1}\\ {\hat{r}}_{2o}^{T}\;J_{2}\\ \vdots \\ {\hat{r}}_{lo}^{T}\;J_{l} \end{array}\right]\dot{q}\leq \left[\begin{array}{c} \phi^{d}{\left\|r_{1o}\right\|}^{2}-\left\|r_{1o}\right\|\\ \phi^{d}{\left\|r_{2o}\right\|}^{2}-\left\|r_{2o}\right\|\\ \vdots \\ \phi^{d}{\|r_{lo}\|}^{2}-\left\|r_{lo}\right\| \end{array}\right],$$ (14)

rewritten, for the $k^{th}$ obstacle, as

$$J_{ok}\dot{q}\;\;\;\leq b_{o}.$$ (15)

Eq. 15 considers the reduced performance capabilities due to nearby obstacles. It is similarly convenient to consider the effects of joint limit proximity in the polytope before transformation to the Cartesian space, thus avoiding improper penalization due to redundancy (Tsai, 1986; Abdel-Malek et al., 2004). For the $i^{th}$ joint, the penalization term is defined as

$$\psi_{i}^{max}=1-{\left(\frac{\text{max}\left({\bar{q}}_{i},q_{i}\right)-{\bar{q}}_{i}}{q_{i}^{max}-{\bar{q}}_{i}}\right)}^{k},\;\;\psi_{i}^{min}=1-{\left(\frac{\text{min}\left({\bar{q}}_{i},q_{i}\right)-{\bar{q}}_{i}}{q_{i}^{min}-{\bar{q}}_{i}}\right)}^{k},$$ (16)

where ${\bar{q}}_{i}$ is given as ${\bar{q}}_{i}=\frac{1}{2}\left(q_{i}^{max}+q_{i}^{min}\right)$, *k* is a positive integer, $\psi_{i}^{max}$ varies from 1 to 0 as the $i^{th}$ joint approaches its limit. Eq. 4 is modified to consider the joint limits

$$\left[\begin{array}{c} I_{n}\\ -I_{n} \end{array}\right]\dot{q}\leq \left[\begin{array}{c} \Psi^{max}\;{\dot{q}}_{max}\\ -\Psi^{min}\;{\dot{q}}_{min} \end{array}\right],$$ (17)

where $\Psi^{max}=diag\left(\psi_{1}^{max}\ldots \psi_{n}^{max}\right)$ and $\Psi^{min}=diag\left(\psi_{1}^{min}\ldots \psi_{n}^{min}\right)$.

By repeating Eq. 15 for *m* obstacles and including the position constraints defined by Eq. 17, the following ℋ-representation of the joint-space polytope, denoted as $Q^{H*}$, is obtained

$$\left[\begin{array}{c} J_{o1}\\ J_{o2}\\ \vdots \\ J_{om}\\ I_{n}\\ -I_{n} \end{array}\right]\dot{q}\leq \left[\begin{array}{c} b_{o1}\\ b_{o2}\\ \vdots \\ b_{om}\\ \Psi^{max}\;{\dot{q}}_{max}\\ -\Psi^{min}\;{\dot{q}}_{min} \end{array}\right].$$ (18)

$Q^{H*}$ can be converted to V-representation using the double description method. The vertex form can then be transformed to the task space using Eq. 1 and in doing so the CMP, denoted as $P^{\text{*}}$ that characterizes the constrained task-space performance, is obtained. The volume of $P^{\text{*}}$, denoted $w_{p}^{\text{*}}$ measures the robot’s velocity capacities while also considering joint position and velocity limits and the constraints imposed by the environment.

### 2.4 Applications of Constrained Manipulability Polytope for Humanoid Robots

In the following, applications for the performance measure are demonstrated using NASA’s humanoid robot Valkyrie (Radford et al., 2015) interacting with a glovebox for nuclear decommissioning task.

#### 2.4.1 Experiment 1: Right Arm Insertion

**FIGURE 1:**
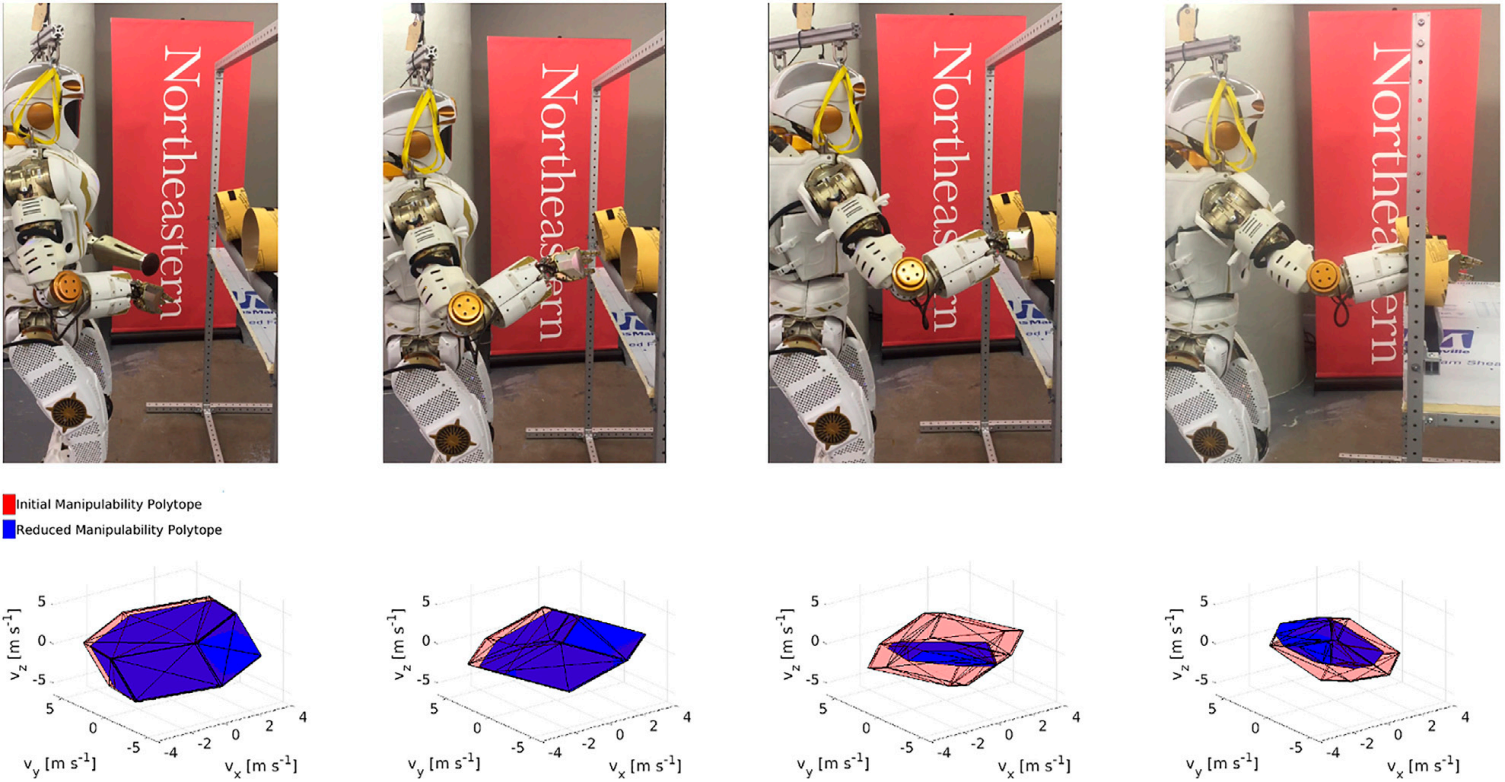
Valkyrie inserting right arm into a glovebox and the evaluation of the manipulability polytope (red) and constrained manipulability polytope (blue) of the right arm. A video of the task is available here: https://www.youtube.com/watch?v=FzlhsLH5IPU.

In the first experiment the right arm of Valkyrie is inserted into the glovebox. The glovebox is considered as an obstacle that reduces the manipulator’s performance, as unwanted collision could be dangerous. Figure 1 shows the right arm insertion task, demonstrating how the CMP changes with time. The initial reduction in manipulability is due to positional joint limits. As the right hand passes through the glovebox port the system experiences a reduction of velocity capacity due to the constrained space signifying that the hand cannot move quickly without increasing the likelihood of a collision. A partial recovery can be observed as the right hand is fully inserted, meaning the system can manipulate objects within the space.

#### 2.4.2 Experiment 2: Dual Arm Insertion

Convex polytopes are geometric objects, thus can be combined through standard geometric operations. These combinations can be used to represent composite robotic chains both serial and parallel. In (Long and Padir, 2019), we have shown how the manipulability of mechanisms in series can be obtained from the *minkowski* sum of sub-mechanisms, while manipulability mechanisms in parallel can be obtained by a straightforward concatenation of inequality constraints.

**FIGURE 2:**
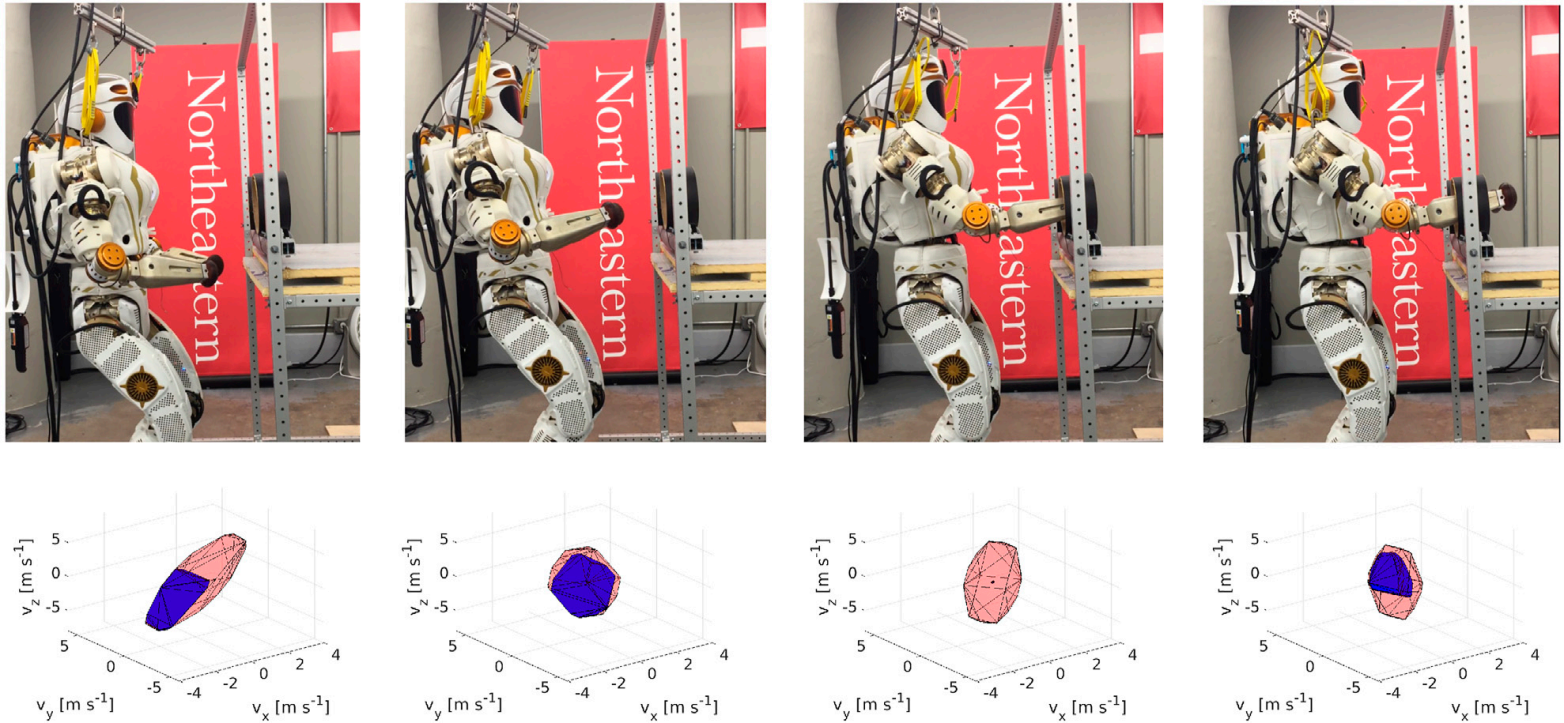
Valkyrie inserting both arms into a glovebox shown at four timesteps along with the coordinating polytopes, $P_{r\cap l}$ and $P_{r\cap l}^{*}$ evaluated at the right end effector. A video of the task is available here: https://youtu.be/1Nouc4f_rIY.

In this experiment, we demonstrate the former, as Valkyrie inserts both arms into the glovebox. It is assumed that the two arms form a closed chain, in a scenario where the arms carry a common object or tool. The goal is to show how the CMP can be combined to obtain that of a closed chain system. To model the closed chain, we use the virtual object procedure, i.e., a rigid straight link extending from the left to the right hand (Long et al., 2015). Figure 2 shows the motion associated with the dual-arm insertion task at four time instants and the CMP for the closed-chain system evaluated at the right-arm end effector. This is obtained by first calculating $P_{r}$ and $P_{l}$, then obtaining the intersection $P_{r\cap l}$, while $P_{r\cap l}^{*}$ is calculated in the same manner. In the third instant $P_{r\cap l}^{*}=\emptyset$, as clearly it is impossible for the arms to enter through individual ports while holding a common object. In contrast, in the fourth instant, $P_{r\cap l}^{*}$ is no longer empty demonstrating the ability to co-manipulate an object within the glovebox.

#### 2.4.3 Experiment 3: Reachability Analysis

**FIGURE 3:**
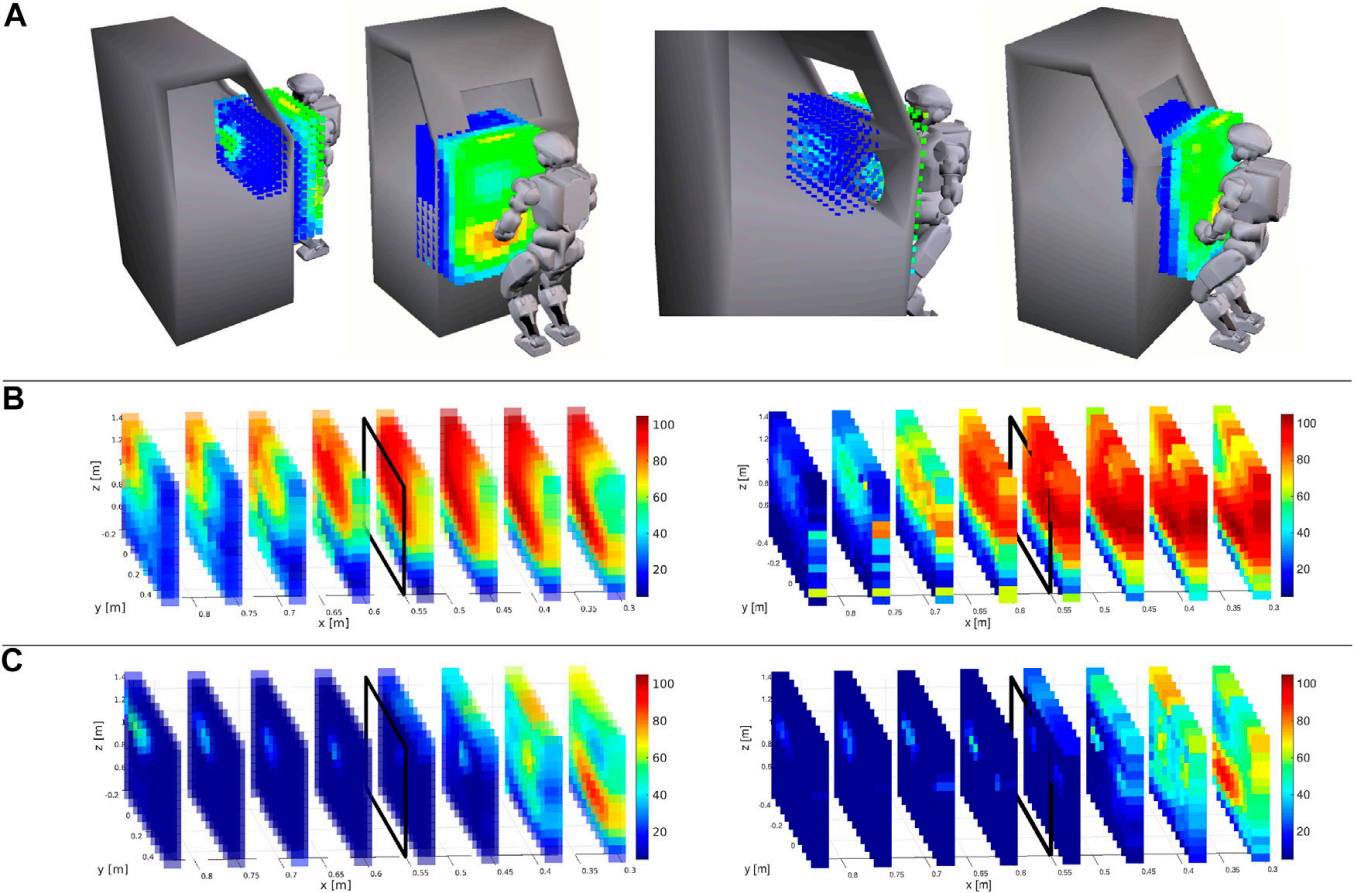
The space is discretized into 3D voxels. **(A)** At each voxel, the IK solution is obtained for the left arm **(left images)** and the right arm **(right images)**. The corresponding volume of the CMP is calculated for each voxel, giving a good understanding of the robot’s workspace. A video is available here: https://youtu.be/jc7X4WakdoE **(B)** The MP’s volume $w_{p}$, i.e, for the left **(left image)** and the right **(right image)** end effectors. The black square shows the location of the glovebox front edge. **(C)** The CMP’s volume $w_{p}^{\text{*}}$ for the left **(left image)** and the right **(right image)** end effectors. High manipulability is possible far from the glovebox, the manipulability is extremely limited once either arm enters the glovebox.

Finally, a reachability study/workspace analysis is presented in Figure 3. The environment is discretized into voxels. At each voxel, an optimization procedure obtains a feasible IK solution while trying to maximize the robot’s distance to obstacles. The CMP is calculated in this configuration. The workspace discretization is shown in Figure 3. The voxel’s color is defined by the volume of $w_{p}^{\text{*}}$, red implys high volume while blue implies empty set. Figure 3B shows the volume of $P_{r}$ and $P_{l}$ along the x−axis, i.e., along the centerline of the glovebox ports, while Figure 3C shows the reduced volume for $P_{r}^{*}$ and $P_{l}^{*}$. The increase in manipulator capacities can be observed as the arms align with the glovebox ports.

## 3 Increasing Stability Using Support Contacts

As nuclear facilities reach the end of their life cycle they must be decommissioned in a safe and efficient manner. A particularly dangerous task is the decontamination of gloveboxes that have been previously used to manipulate radioactive material. Although a robotic system that is specifically designed for glovebox operations may be the best solution, humanoid robots are an attractive option since they can operate in a variety of environments and use tools that are designed for humans. While conducting operations within the glovebox, the constraints imposed by the ports, gloves, and the external structure, which effectively fix the arms at the entry points, must be considered. The inability to alter body configuration greatly diminishes the robot’s capacity to take steps in arbitrary directions. This in turn leads to a danger of toppling during task execution as the system cannot easily change the support polygon’s location.

Toppling occurs when the ZMP leaves the support polygon (SP) (Vukobratović and Borovac, 2004). If the SP cannot be displaced, alternative methods to maintain stability must be employed. For example, in (Rasheed et al., 2018), the ZMP position for a cable-driven mobile robot is modified online by a tension distribution algorithm. In (Khatib and Chung, 2014), it is shown that the SP size can be increased by using supplementary contact points. Similar to these approaches, we propose to exploit the contacts in the glovebox (i.e., leaning on the entry ports) in order to shift the ZMP towards the center of the SP while performing manipulation tasks.

This section presents our work on planning kinematic motions with support contacts without a predefined contact schedule that maintains the stability of the ZMP. To accomplish this, we model rigid-body contacts using complementarity constraints and solve a nonlinear constrained optimization for joint velocities and contact forces. Owing to the differentiable contact model, gradient-based optimization can reason about contacts between the robot arms and the glovebox ports. This optimization also respects constraints that ensure an object is grasped by the end effectors, the ZMP is in a safe region, and the deviation of the object’s position from a desired position is acceptable. Furthermore, we present a null-space-based torque controller that prioritizes the stability, i.e., generating the support forces, and projects the torques needed for the manipulation task onto the null space of the support forces. The proposed methodology is tested through 2.5D, quasi-static simulations by considering a humanoid robot with two planar arms manipulating a relatively heavy object on an elevated plane representing the glovebox.

### 3.1 Related Work

For planning a motion with contact interactions, both the discrete contact events (e.g., making/breaking contacts at certain locations) and the continuous variables (e.g., joint positions, contact forces, stability constraints) must be considered. One approach is to use a contact-before-motion planner such as those presented in (Hauser et al., 2005; Escande et al., 2013). In this case, first, a sequence of contacts at predefined locations is determined, then a continuous motion is planned subject to contact constraints. In contrast, in a motion-before-contact planner, the contacts are obtained as a result of the motion planning (Escande et al., 2013). Alternatively, contact-implicit motion planning (also known as, motion planning through contacts) can be used to plan for smooth motions and contact events at the same level.

In contact-implicit motion planning, a differentiable contact model is used to enable gradient-based optimization to reason about contacts. Complementarity constraints are widely used to model rigid-body contacts with friction, as proposed in (Stewart and Trinkle, 1996; Anitescu and Potra, 1997). (Yunt and Glocker, 2005; Posa et al., 2014) use complementarity constraints to model contacts in a trajectory optimization problem. The main idea here is to consider the contact-related parameters as additional optimization variables such that the contact events evolve along with continuous motion variables. Such an optimization problem can be solved locally through constrained nonlinear optimization algorithms such as sequential quadratic programming (Anitescu and Potra, 1997; Fletcher and Leyffer, 2004).

Once a kinematic motion with contact interactions is planned, it can be executed using a null-space-based controller. Park and Khatib (Park and Khatib, 2006) proposed a torque control framework for humanoid robots with multiple contacts and verified the method experimentally in (Park and Khatib, 2008). Moreover, they extended this method to a unified hierarchical whole-body control framework for humanoid robots in (Khatib et al., 2008). In this framework, tasks are hierarchically ranked. Thus, the torques required for a lower-priority task are projected onto the null-space of the Jacobian matrix associated with a higher-priority task. In (Henze et al., 2016a), a whole-body torque controller for humanoid robots is proposed that combines passivity-based balancing proposed in (Henze et al., 2016b) with a hierarchical null-space-based control that is similar to (Khatib et al., 2008).

### 3.2 Methodology

#### 3.2.1 Static Equilibrium

For being balanced, the robot needs to be in static equilibrium (Vukobratović and Borovac, 2004). In this case, the static equilibrium of the system can be evaluated considering the following wrenches: the wrench due to the robot’s mass, the wrenches at the end effectors due to the object wrench, and the wrenches at the support contact points. Henceforward, we enumerate the left and right arms as the first and second arms, respectively.

In the static equilibrium, the net force must be zero:

$$\sum f=0\Rightarrow f_{r}+mg+\sum_{i=1}^{2}f_{s_{i}}+\sum_{i=1}^{2}f_{c_{i}}=0,$$ (19)

where *m* is the total mass of the robot, $g≜{\left[0,0,-g\right]}^{T}$ is the gravity vector, $f_{s_{i}}\in {\mathbb{R}}^{3}$ is the force at the support point between the $i^{th}$ arm and the glovebox port, $f_{c_{i}}\in {\mathbb{R}}^{3}$ is the force at the contact point between the object and the $i^{th}$ end effector, and $f_{r}\in {\mathbb{R}}^{3}$ is the ground reaction force.

Additionally, the projection of the net moment, M onto the horizontal xy plane must be zero, i.e., $M^{x}=0$ and $M^{y}=0$:

$$\sum M^{H}=0\Rightarrow {\left(p_{r}\times f_{r}\right)}^{H}+{\left(p_{CoM}\times mg\right)}^{H}+\sum_{i=1}^{2}{\left(p_{s_{i}}\times f_{s_{i}}+M_{s,i}\right)}^{H}+\sum_{i=1}^{2}{\left(p_{c_{i}}\times f_{c_{i}}+M_{c,i}\right)}^{H}=0,$$ (20)

where $a^{H}$ denotes the horizontal projection of a vector a, $p_{r},p_{CoM},p_{s_{i}},p_{c_{i}}\in {\mathbb{R}}^{3}$ are the positions of the ground reaction force (i.e., the ZMP), the robot’s center of mass (CoM), the support points on the glovebox ports and the contact points on the object, with respect to the world frame. $M_{s,i},M_{c,i}\in {\mathbb{R}}^{3}$ are the moments at the support and grasp points. The position of the ZMP, $p_{r}$, is obtained by solving (Eq. 19, 20) simultaneously. In order to avoid toppling, the ZMP must lie in the support polygon (SP), namely, the convex hull of the robot’s feet. The object wrench $h_{o}\in {\mathbb{R}}^{6}$ can be obtained in terms of the wrenches applied by the end effectors as follows (Caccavale and Uchiyama, 2016):

$$h_{o}=\left[\begin{array}{cc} W_{c_{1}} & W_{c_{2}} \end{array}\right]\left[\begin{array}{c} h_{c_{1}}\\ h_{c_{2}} \end{array}\right]=Wh_{c},$$ (21)

using the wrench matrix $W_{c_{i}}\in {\mathbb{R}}^{6\times 6}$ that transforms the wrench at the $i^{th}$ contact point, $h_{c_{i}}\in {\mathbb{R}}^{6}$, to the wrench at the origin of the object frame, that is the center of the object in this case, and given by:

$$W_{c_{i}}=\left[\begin{array}{cc} I_{3} & 0_{3}\\ -{\hat{r}}_{c_{i}} & I_{3} \end{array}\right],$$ (22)

where ${\hat{r}}_{c_{i}}$ is the skew-symmetric matrix representation of the vector from the $i^{th}$ contact point $c_{i}$ to the origin of the object frame, and $I_{3}$ and $0_{3}$ are 3×3 identity and zero matrices. Then, given the object wrench, the wrenches at the end effectors can be calculated from $h_{c}=W^{+}h_{o}$, where $W^{+}$ is the Moore-Penrose pseudo-inverse of the matrix $W\in {\mathbb{R}}^{6\times 12}$.

#### 3.2.2 Motion Planning

In this work, we ignore the dynamic effects and investigate the quasi-static case for dual-arm manipulation of an object in a confined space, i.e., a glovebox. In the following, robot’s joint positions and velocities are denoted by q and $\dot{q}$, while those of the $i^{th}$ arm are referred to as $q_{i}$ and ${\dot{q}}_{i}$. The objective is to preserve the robot’s balance during the manipulation task. In other words, our goal is to find the joint positions that would keep the robot’s ZMP in a safe region by leaning on the glovebox ports while simultaneously maintaining the manipulated object’s desired position. For this purpose, we form a nonlinear constrained optimization and solve it for the joint displacements and the support contact forces.

In order to take into account the rigid-body contacts between the robot and the glovebox ports, we use the following complementarity constraints, as in (Anitescu and Potra, 1997; Posa et al., 2014):

ϕ(q)≥0, (23a)

γ≥0, (23b)

$$\gamma^{T}\phi \left(q\right)=0,$$ (23c)

where $\phi \left(q\right)\in {\mathbb{R}}^{n_{p}}$ is the vector of signed distance for $n_{p}$ contact pairs, i.e., each pair comprises of a robot’s link and a contact candidate in the environment; and $\gamma \in {\mathbb{R}}^{n_{p}}$ is the vector of the magnitude of normal support force. (Eq. 23a) prevents any interpenetration, (Eq. 23b) ensures that the bodies can only push each other, and (Eq. 23c) allows force generation only when bodies are in contact. Thus, only one of these variables (either ϕ(q) or γ) can be non-zero for a given time and contact pair. We relax the complementarity condition (Eq. 23c) by converting it into an inequality constraint through a slack variable and penalizing the slack variable in the cost so that potential numerical issues are mitigated, as described in (Fletcher and Leyffer, 2004; Manchester and Kuindersma, 2017). For numerical efficiency, the complementarity constraints, including the relaxation, are evaluated elementwise, i.e., separately for each contact pair.

As a result, the following optimization problem is solved for the joint displacements $\text{Δ}q≜q_{k+1}-q_{k}$, the magnitudes of the normal contact forces at the support points γ, and the slack variables s:

$$\underset{\Delta q,\gamma ,s}{\text{minimize }}\;\;w_{1}{\left\|p_{o}^{e}\right\|}^{2}+w_{2}{\left\|\Delta q\right\|}^{2}+w_{3}{\left\|s\right\|}^{2}$$ (24a)

subject to:

ϕ(q),γ,s≥0, (24b)

$$\gamma^{T}\phi \left(q\right)\leq s,$$ (24c)

$$c_{g}\left(q\right)=0,$$ (24d)

$$\left\|p_{r}^{d}-p_{r}\right\|\;\;\leq r_{s},$$ (24f)

$$\left\|p_{o}^{e}\right\|\;\;\leq r_{o},$$ (24g)

where ‖⋅‖ is the Euclidean norm, $w_{i}$ is the weight (a positive scalar) associated with the $i^{th}$ term of the cost function, $p_{o}^{e}$ is the deviation of the object’s position from the desired position, $c_{g}\left(q\right)=0$ ensures that the end effectors are grasping the object, $p_{r}^{d}$ is the desired position of the ZMP (i.e., the center of the SP), and $r_{s}$ and $r_{o}$ are the radii of the safe circle (SC) for the ZMP and the admissible sphere for the object position.

#### 3.2.3 Torque Control

Using this optimization procedure, the robot configuration and the support forces’ magnitude are obtained. Nevertheless, a torque controller is necessary to execute the planned motions.

The torques necessary to generate the desired object wrench $\tau_{h}$ can be obtained as:

$$\tau_{h}=\left[\begin{array}{cc} J_{1}^{T} & 0\\ 0 & J_{2}^{T} \end{array}\right]W^{+}h_{o}=J^{T}W^{+}h_{o}$$ (25)

$J_{i}\in {\mathbb{R}}^{3\times 4}$ is the kinematic Jacobian matrix that maps the joint velocities to the translational end-effector velocities for the $i^{th}$ arm.

The support forces are oriented normal to the contacting robot geometry. Hence, using the contact angle $\beta_{i}$ and the normal force magnitude γ, the support force for the $i^{th}$ arm can be calculated as:

$$f_{s_{i}}={\left[\begin{array}{ccc} \gamma_{i}cos\left(\beta_{i}\right) & \gamma_{i}sin\left(\beta_{i}\right) & 0 \end{array}\right]}^{T}.$$ (26)

Similarly, the joint torques required to generate these forces, denoted as $\tau_{s}$, can be calculated as described in (Park and Khatib, 2008). In our case, there is a maximum of two supports points at a given time, therefore:

$$\tau_{s}=\left[\begin{array}{cc} J_{s_{1}}^{T} & 0\\ 0 & J_{s_{2}}^{T} \end{array}\right]\left[\begin{array}{c} f_{s_{1}}\\ f_{s_{2}} \end{array}\right]=J_{s}^{T}f_{s}.$$ (27)

$J_{s_{i}}\in {\mathbb{R}}^{3\times 4}$ is the Jacobian matrix that maps the joint velocities of the $i^{th}$ arm to the translational velocities at the support point.

For the glovebox task, the support forces are crucial to maintain the stability of the robot, while generating the desired object wrench has a lower priority. Thus, we compose the joint torques, τ such that the manipulation torques $\tau_{h}$ are projected onto the null space of the stability torques:

$$\tau =\tau_{s}+N_{s}\tau_{h},$$ (28)

where

$$N_{s}=I_{n_{d}}-J_{s}^{T}\left(J_{s}^{+T}\right)$$ (29)

is the null space projector of the support forces, and $n_{d}$ is the DOF of the whole robot. Consequently, the resulting joint torques would generate the desired support forces to ensure the balance of the robot and create an object wrench using the redundancy of the robot.

### 3.3 Results

To test the proposed framework, we run simulation experiments in which a humanoid robot that has two planar 4-DOF arms with revolute joints manipulates a relatively heavy rigid bar on an elevated plane. The robot’s arms pass through two ports representing the glovebox. We neglect the dynamics (i.e., velocities and accelerations) and assume point contacts without friction. The weights are selected as $w_{1}=10^{3}$, $w_{2}=10^{2}$, and $w_{3}=10^{6}$. The initial values for all the decision variables are zero. The radii of tolerance circles for the ZMP and the object position are selected as $r_{s}=0.15$ m and $r_{o}=0.1$ m. The masses of the robot and the object are 54 kg and 12 kg. The desired motion of the object is in +y direction; hence, the desired object wrench to generate an acceleration in this direction is given as $h_{o}={\left[0,10,-117.72,0,0,0\right]}^{T}$.

**FIGURE 4:**
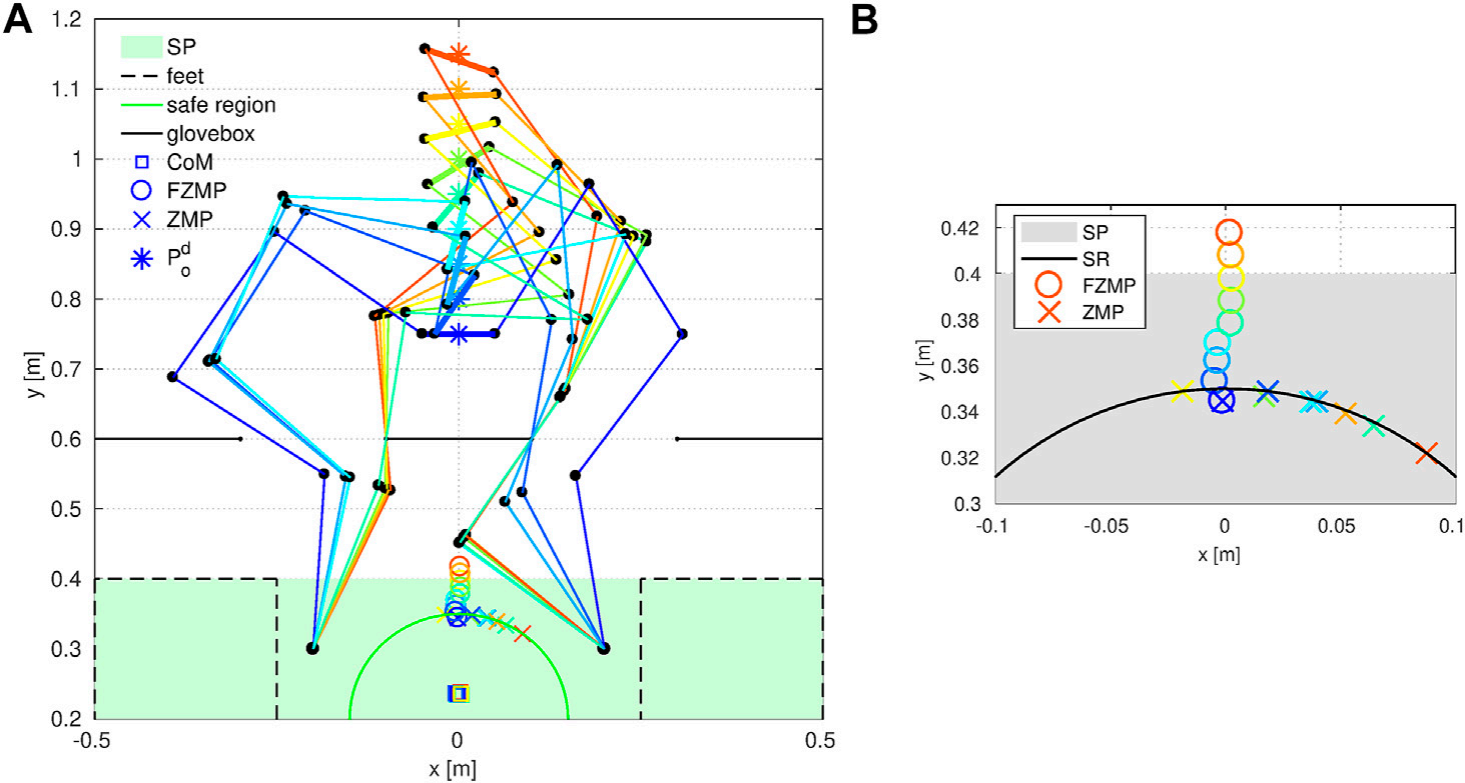
**(A)** Planned motion for carrying an object on a straight path. **(B)** Zoomed in change of the ZMP and the FZMP (i.e., fictitious ZMP without supporting contacts). throughout the task.

We investigate the task of moving the object 40 cm forward on a straight path that consists of nine equally spaced waypoints. The results are depicted in Figure 4. Each step of the motion is indicated by a color from blue to red. In the initial configuration (indicated by blue), the robot grasps the object from both ends. During the simulation, the position of the ZMP is calculated with and without the effect of the support contacts on the glovebox frame. The latter is known as the fictitious ZMP (FZMP) (Vukobratović and Borovac, 2004) and may fall outside of the SP.

The results show that the contact-implicit motion planning method can increase the stability of the robot using support contacts while performing the manipulation task. The robot makes contacts with the glovebox to maintain its balance while moving the object on the desired path. As soon as the FZMP leaves the SR in the second step, the right arm makes a contact with the left end of the port to push the ZMP into the SR. As the object moves further away from the base, the contact angle is varied so that the magnitude of the support force in −x-direction is larger. This is required due to the circular shape of the SR. However, as object moves further from the base, simply changing the contact angle is no longer sufficient, thus the left arm also makes contact with the right end of the left port. As a result, the object is successfully transported along the desired path with a position error of 0.1 m (i.e., the allowed deviation) in each step after the initial configuration.

A zoomed-in version of the SP area is depicted in Figure 4B to show the change of the ZMP and the FZMP throughout the simulation. Even though the FZMP moves forward along with the object’s position and leaves the SP eventually, the ZMP does not leave the SR owing to the support forces. It is also noted that, in Step 7 (indicated by yellow), the ZMP is more centralized with respect to the *y* axis compared to the other steps due to the symmetry of the support forces.

**FIGURE 5:**
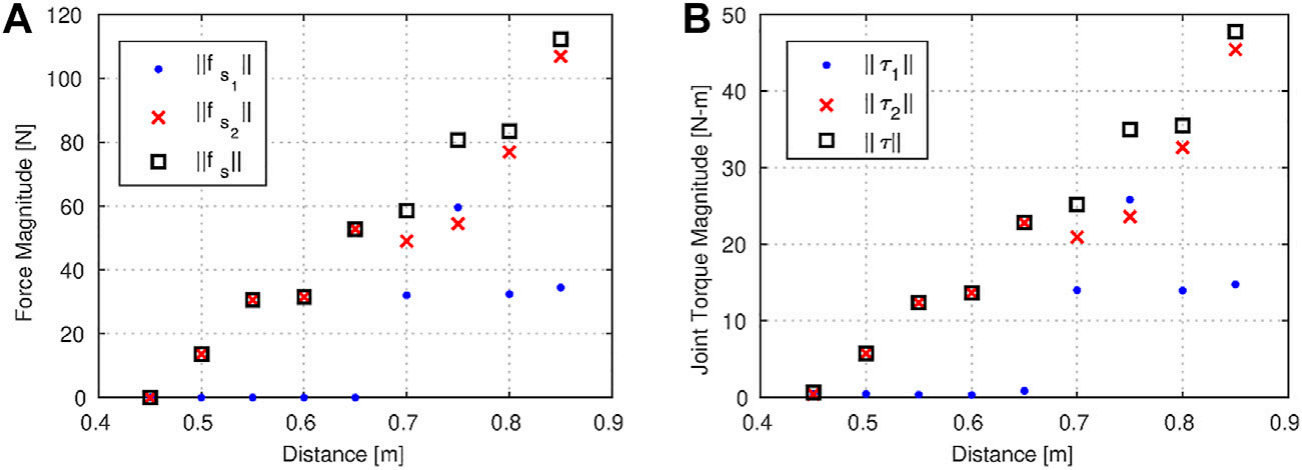
The magnitudes of **(A)** the support forces vs. the distance of the object from the base, and **(B)** the joint torques vs. the distance of the object from the base.

Figure 5 show the magnitudes of the support forces and the joint torques with respect to the distance between the object and the robot’s base. The magnitudes of the support forces are much larger than the magnitude of the object wrench, and therefore the torques are much more affected by the support forces than the object wrench. This is why force and torque vs. distance characteristics are quite similar—i.e., the torque is dominated by the support forces (especially after Step 5). Moreover, the changes of $\left\|f_{s}\right\|$ and ‖τ‖ with the distance are almost linear, as one may anticipate. Apart from this, the magnitude of the support force on each arm is quite similar to each other in Step 7, as consistent with the observation regarding the more centralized ZMP in this step. Except for Steps 1 and 7, the magnitude of the support force on the right arm is always bigger than the one on the left arm, which shifts the ZMP in +x direction. Such an unbalanced distribution of forces might be undesirable since higher joint torque limits would be required. Thus, enforcing a more uniform distribution of the support forces may be a future work.

## 4 Dynamic Non-prehensile Manipulation

There is an increasing need to carry out decontamination and decommissioning tasks in safe and effective manner. A particularly dangerous task is glovebox decontamination and decommissioning that typically involves transporting debris and objects from the interior of the glovebox to an exit port, where they are bagged and removed (Long et al., 2018; Önol et al., 2018). Such tasks do not always require dexterous manipulation behaviors and instead simply require objects to be push from the interior to the exit port of a glovebox.

Contact-implicit trajectory optimization (CITO) is a promising method to generate contact-rich behaviors given only a high-level definition of a task. In this approach, a differentiable contact model is used to enable gradient-based optimization to reason about contacts such that discrete contact events and continuous trajectories are found simultaneously as a result of smooth optimization.

In this section, we present a CITO method based on a variable smooth contact model to plan dynamic non-prehensile manipulation behaviors for a 7-DOF robot arm in a highly-constrained environment. We demonstrate that the proposed method can solve complex tasks despite tight constraints imposed by the environment by exploiting the smooth virtual forces. Moreover, we experimentally verify that the physical inaccuracy introduced by the residual virtual forces is admissible and the motions found by this framework are realistic enough to be run on the hardware.

### 4.1 Related Work

Complementarity constraints are widely used to model rigid-body contacts in trajectory optimization (Yunt and Glocker, 2005; Posa et al., 2014; Gabiccini et al., 2018). This approach can find complex motions, but it typically suffers from poor convergence speed. Thus (Tassa et al., 2012; Mordatch et al., 2015; Mastalli et al., 2016; Neunert et al., 2016; Manchester and Kuindersma, 2017), use smoother fragments of the complementarity constraints. (Neunert et al., 2017; Giftthaler et al., 2017; Marcucci et al., 2017; Neunert et al., 2018), on the other hand, define contact forces as a smooth function of distance, i.e., a smooth contact model. Using such a contact model, highly-dynamic complex motions for a quadruped robot are planned and executed in real-time in (Neunert et al., 2018). However, it is difficult to tune smooth contact models (Carius et al., 2018), and the resulting motions may be physically inaccurate due to the non-physical contact forces that act from distance. In order to address these problems, we have recently proposed a variable smooth contact model (VSCM) (Önol et al., 2018) that injects virtual forces to the underactuated dynamics with frictional rigid-body contact mechanics, such that the states of the manipulator and the objects are coupled in a smooth way. Furthermore, the smoothness of the contact model is adjusted by optimization such that large virtual forces are permitted in the initial phases of optimization but vanish as the optimization converges. As a result, the VSCM improves the convergence of CITO without compromising the physical fidelity of resulting motions.

CITO has been used for animated characters (Mordatch et al., 2021a; Mordatch et al., 2012b) and in robotics (Tassa et al., 2012; Posa et al., 2014; Mordatch et al., 2015; Mastalli et al., 2016; Manchester and Kuindersma, 2017; Neunert et al., 2017; Carius et al., 2018; Neunert et al., 2018; Winkler et al., 2018). Although this method is task independent and can be generalized to both locomotion and manipulation problems, the majority of the related literature considers only the former. On the other hand, in (Mordatch et al., 2012a; Posa et al., 2014; Gabiccini et al., 2018), manipulation tasks are investigated but their analyses are either limited to a planar case or based on animated characters where physical fidelity is not critical. Recently (Önol et al., 2018, 2019, 2020; Sleiman et al., 2019), used CITO for non-prehensile manipulation tasks. Yet, they consider only tabletop pushing scenarios. Moreover, in general, experimental results in this domain are very limited, albeit with some notable exceptions (Mordatch et al., 2015; Mastalli et al., 2016; Neunert et al., 2017, 2018; Giftthaler et al., 2017; Carius et al., 2018; Winkler et al., 2018; Sleiman et al., 2019). Nonetheless, to the best of our knowledge, there is no experimental verification of CITO for constrained dynamic manipulation.

### 4.2 Methodology

#### 4.2.1 Dynamic Model

The dynamics of an underactuated system consisting of an $n_{a}$-DOF manipulator and $n_{u}$-DOF objects that are subject to frictional rigid-body contacts and virtual forces is given by

$$M\left(q\right)\ddot{q}+c\left(q,\dot{q}\right)=S_{a}^{T}\tau +J_{c}^{T}\left(q\right)\lambda_{c}+S_{u}^{T}\lambda_{v},$$ (30)

where $q≜{\left[q_{a}^{T},q_{u}^{T}\right]}^{T}\in {\mathbb{R}}^{n_{a}+n_{u}}$ is the configuration vector; $M\left(q\right)\in {\mathbb{R}}^{\left(n_{a}+n_{u}\right)\times \left(n_{a}+n_{u}\right)}$ is the mass matrix; $c\left(q,\dot{q}\right)\in {\mathbb{R}}^{n_{a}+n_{u}}$ is the bias term comprising of the Coriolis, centrifugal, and gravitational effects; $S_{a}=\left[{\mathrm{II}}_{n_{a}\times n_{a}}\;0_{n_{a}\times n_{u}}\right]$ is the selection matrix for the actuated DOF and $S_{u}=\left[0_{n_{u}\times n_{a}}\;{\mathrm{II}}_{n_{u}\times n_{u}}\right]$ is the selection matrix for the unactuated DOF; $\tau \in {\mathbb{R}}^{n_{a}}$ is the vector of generalized joint forces; $\lambda_{c}\in {\mathbb{R}}^{6n_{c}}$ is the vector of generalized contact forces at $n_{c}$ external contact points and $J_{c}\left(q\right)\in {\mathbb{R}}^{6n_{c}\times \left(n_{a}+n_{u}\right)}$ is the Jacobian matrix mapping the joint velocities to the Cartesian velocities at the contact points, and $\lambda_{v}\in {\mathbb{R}}^{n_{u}}$ is the vector of generalized virtual forces on the unactuated DOF. For $n_{f}$ free bodies in SE (Eq. 3) (e.g., objects), $n_{u}=6n_{f}$. The state of the system is represented by $x≜{\left[q^{T}\;{\dot{q}}^{T}\right]}^{T}\in {\mathbb{R}}^{n}$ where $n=2\left(n_{a}+n_{u}\right)$.

In this study, τ is decomposed as $\tau =\tau_{u}+\tilde{c}$, where $\tilde{c}\in {\mathbb{R}}^{n_{a}}$ is an estimation of the non-zero part of $S_{a}^{T}c\left(q,\dot{q}\right)$ and $\tau_{u}\in {\mathbb{R}}^{n_{a}}$ is the vector of control variables in terms of generalized joint forces. As a result, the control term $\tau_{u}$ is linearly related to the joint accelerations in the absence of external contact.

#### 4.2.2 Contact Model

**FIGURE 6:**
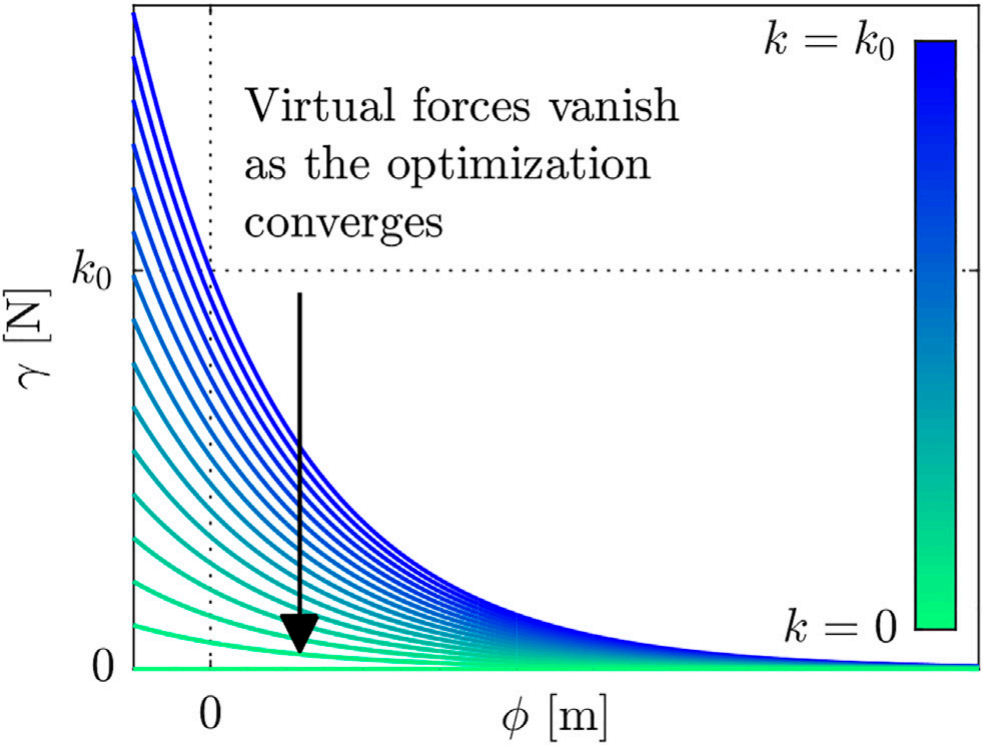
The relationship between the virtual force and the distance. $k_{0}$ represents the initial value of the virtual stiffness *k*, and the arrow shows the evolution of the virtual forces throughout optimization.

The virtual forces generated by the contact model acts upon the unactuated DOF in addition to the external rigid-body contacts. Consequently, the robot’s and objects’ dynamics are related through the virtual forces. We assume an exponential relationship between the magnitude of the normal contact force *γ* and the signed distance between paired contact geometries *ϕ*, as depicted in Figure 6. While frictional forces are not considered in this contact model, the rigid-body contact mechanics [i.e., $\lambda_{c}$ in (Eq. 30)] are frictional. Hence, the resulting motions include frictional contacts once the virtual forces vanish.

For the $i^{th}$ contact candidate, the magnitude of the normal contact force is calculated from the virtual stiffness $k_{i}$ and $\alpha_{i}$ that determines the curvature:

$$\gamma_{i}\left(x\right)=k_{i}e^{-\alpha_{i}\phi_{i}\left(x\right)}.$$ (31)

This model is analogous to a spring model and (Marcucci et al., 2017) lists several reasons for not using damping (i.e., a velocity term) in such a contact model. The corresponding virtual force effective at the center of mass of the free body associated with the contact candidate $\lambda_{v,i}\in {\mathbb{R}}^{6}$ is:

$$\lambda_{v,i}\left(x\right)=\gamma_{i}\left(x\right)\left[\begin{array}{c} {\mathrm{II}}_{3}\\ {\hat{l}}_{i} \end{array}\right]n_{i}\left(x\right),$$ (32)

where ${\mathrm{II}}_{3}$ is 3×3 identity matrix; $l_{i}$ is the vector between the end effector and the center of mass of the object that is associated with the contact candidate; ${\hat{l}}_{i}$ is the skew-symmetric matrix form of $l_{i}$; and $n_{i}\in {\mathbb{R}}^{3}$ is the unit vector that is normal to the contact surface on the object. Hence, the net virtual force on an object is the sum of the virtual forces associated with the contact candidates on that object.

In the variable smooth contact model, the virtual stiffness *k* for each time step and contact pair is a decision variable of optimization and initialized with a large value such that there is a non-zero virtual force on each contact candidate. Nonetheless, the virtual forces are penalized as an integrated cost, so that they vanish as the optimization converges, see Figure 6. This approach helps to discover contact candidates that are initially distant.

#### 4.2.3 Trajectory Optimization

In this study, the optimal control problem is transcribed into a finite-dimensional nonlinear constrained optimization by assuming constant control inputs over *N* discretization intervals. Final cost terms penalize the deviations of the objects’ poses from desired poses, $p_{o}^{e}$ and $\theta_{o}^{e}$. Integrated cost terms are defined in terms of the velocities $\dot{x}$ and the virtual forces γ. As a result, the final and integrated components of the cost ($c_{F}$ and $c_{I}$) are calculated in terms of the weights $w_{1,\ldots ,4}$, the control sampling period $t_{c}$ by:

$$c_{F}=w_{1}{\left\|p_{o}^{e}\right\|}^{2}+w_{2}{\left\|\theta_{o}^{e}\right\|}^{2},$$ (33a)

$$c_{I}=t_{c}\overset{N}{\underset{i=1}{\sum }}\left(w_{3}{\left\|{\dot{x}}_{i}\right\|}^{2}+w_{4}{\left\|\gamma_{i}\right\|}^{2}\right).$$ (33b)

The following optimization problem is solved by a sequential quadratic programming (SQP) algorithm by rolling out the dynamics:

$$\begin{array}{l} \text{minimize}\;\;\;c_{F}+c_{I}\\ \;\;\;\;\;\;\;\;\;\;\;\;\;\;\;\;\;\;\;\;\;\;\tau_{u,1,\ldots ,N},k_{1,\ldots ,N} \end{array}$$ (34a)

$$\text{subject to}:\;\tau_{u,L}\leq \tau_{u,1,\ldots ,N}\leq \tau_{u,U},\;0\leq k_{1,\ldots ,N}\leq k_{0}.$$ (34b)

The lower and upper bounds for the control variables $\tau_{u,L}$ and $\tau_{u,U}$ are determined from the torque limits of the robot. However, it is noted that the bias in the torque decomposition, $\hat{c}$, is not considered while setting the torque limits. The virtual stiffness variables are bounded above by their initial values $k_{0}$, which is selected as a large value to facilitate convergence.

### 4.3 Experiments

#### 4.3.1 Application: Non-Prehensile Manipulation in a Glovebox

**FIGURE 7:**
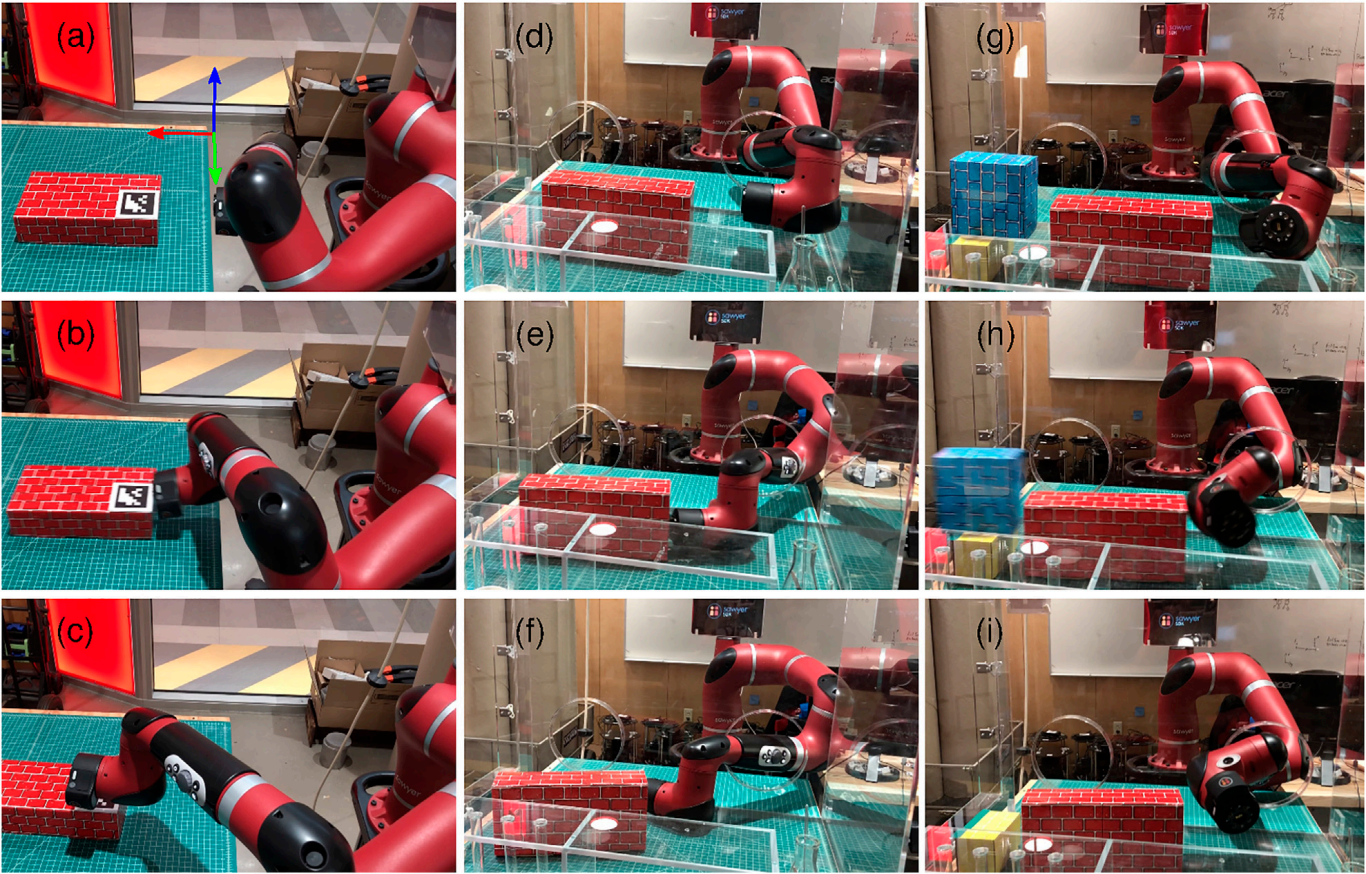
Snapshots from the hardware experiments: pushing an object on a table **(A–C)**, pushing an object within a glovebox **(D–F)**, manipulating an unreachable object by exploiting inter-object contacts **(G–I)**.

Our overall objective is to enable a robot to carry out such manipulation tasks with only high-level commands such as desired object poses. Figure 7D shows Sawyer, a 7-DOF robot arm, and a mock-up glovebox environment. Non-prehensile manipulation is advantageous in this case, as the highly-constrained environment means that grasp configurations are difficult to obtain. Thus, we consider non-prehensile manipulation tasks in a glovebox.

The proposed method is tested in three different scenarios of increasing complexity: 1) pushing an object on a table, Figure 7A-C 2) pushing an object in a glovebox, Figure 7D-F, and 3) ejecting an unreachable object from the glovebox by exploiting physical interactions in the environment, Figure 7G-I. In the first case, there is a (red) box on a table and the task is to move it 20 cm along the *x* axis, see Figure 7A for the reference frame. In the second case, the task is to move the object 10 cm along the −y axis in the glovebox, Figure 7D. In the last case, two boxes that are placed next to each other are considered, as shown in Figure 7G, and the task is to eject the one that is further away from the robot (i.e., the blue box) from the glovebox. In other words, this task requires moving the blue box at least 15 cm along the −y axis so that it will leave the glovebox through the exit port. In all cases, the desired rotation is zero.

#### 4.3.2 Experimental Setup

In the experiments, standard and commercially-available hardware^2^ is used in order to facilitate reproducibility. The dynamics is modeled using MuJoCo physics engine (Todorov et al., 2012) with time steps of 5 ms while the control sampling period is 50 ms. The SQP solver SNOPT (Gill et al., 2005) is used to solve the optimization problem. As an interface between MuJoCo and SNOPT, IFOPT (Winkler et al., 2018) is employed. The planned position, velocity, and acceleration trajectories are interpolated with 10 ms steps and executed on the robot by using the built-in inverse dynamics feed-forward controller of Sawyer. For detecting the poses of the objects and the glovebox through the head camera of Sawyer, AprilTag 2 algorithm (Wang and Olson, 2016; Malyuta, 2017) is used.

For all cases, the weights are $w_{1}=10^{3}$, $w_{2}=10^{3}$, $w_{3}=1$, and $w_{4}=1$. The initial trajectory is set at zero torque values. The initial value and the upper bound for the virtual stiffness is 5 N/m for the red box and 1 N/m for the blue box, since the blue box is lighter than the red box. *α* in Eq. 31 is selected such that $\gamma =k_{0}\times 10^{-2}$ for each contact candidate in the initial configuration. Namely, the optimization is started with a trivial initial guess in which the robot stands still for the whole simulation, and there is no heuristic regarding the contact interactions for any task.

### 4.4 Results

Figure 7 demonstrates initial, intermediate, and final snapshots from the experiments. Table 1 shows the position and orientation deviations for the object ($\left\|p_{o}^{e}\right\|$ and $\left\|\theta_{o}^{e}\right\|$) for the simulation and hardware experiments and the discrepancy between them. Additionally, the physical inaccuracy caused by the residual virtual forces $\psi =t_{c}\underset{i}{\sum }\left\|\gamma_{i}\right\|$ is shown for the simulations. For the last case, only $\delta \left\|p_{o}^{e}\right\|$ and $\delta \left\|\theta_{o}^{e}\right\|$ for the red box are shown since the blue box is ejected (i.e., could not be tracked) and there is no desired pose for the red box.

**TABLE 1:** Numerical results from simulation and hardware experiments for all cases.

Task	Simulation			Experiment		Discrepancy	
	*ψ* [N-s]	$\left\|p_{o}^{e}\right\|$ [m]	$\left\|\theta_{o}^{e}\right\|$ [rad]	$\left\|p_{o}^{e}\right\|$ [m]	$\left\|\theta_{o}^{e}\right\|$ [rad]	$\left\|p_{o}^{e}\right\|$ [m]	$\left\|\theta_{o}^{e}\right\|$ [rad]
1	0.8847	0.0025	0.2772	0.0495	0.0112	0.0470	0.2660
2	1.0698	0.0085	0.1222	0.0777	0.3404	0.0692	0.2182
3	0.1466	N/A		N/A		0.0336	0.1562

Despite the trivial initial guess and no additional tuning for different tasks, the proposed method is capable of finding a motion that successfully completes each task in simulation. That is, the object is moved to within 1-cm radius of the desired position while the change of orientation is negligible (i.e., smaller than 15°). It is noteworthy that in the second and third cases, the glovebox port imposes tight constraints on the motion and the workspace of the robot, yet still our method can handle this without any additional penalties or constraints for collisions with the glovebox or tuning. Moreover, the last task requires the blue box to have a high velocity when the contact between it and the red box is broken because when the red box collides with the yellow box that is under the blue box, the red box cannot apply a force on the blue box anymore and the blue box would still be in the glovebox. Thus, using a non-dynamic planner or running the resulting motions through a position controller without velocities and accelerations would not work in this case.

A more detailed numerical analysis of the experiments is given in the following. In the simulation for Task 1, the box is moved 19.8 cm along the *x* axis and 0.7 cm along the *y* axis; whereas, in the hardware experiment, the box is pushed only 15 cm along the *x* axis and 0.5 cm along the *y* axis. For Task 2, the displacements of the box along the −*y* and *x* axes are 9.8 and 0.8 cm in the simulation. In the experiment, the box is moved 8 cm along the −*y* axis, which is satisfactory, but also 7 cm along the −*x* axis due to the relatively large rotation (ca. 20°) about the *z* axis. In Task 3, the blue box is ejected from the glovebox, namely the task is completed, both in the simulation and the hardware experiment. Since the final position of the blue box could not be detected in the experiment, only the final positions of the red box are compared here. In the simulation, the red box is moved 10.8 cm along the −*y* axis and 0.4 cm along the −*x* axis, and these quantities are 7.5 and 0.3 cm for the hardware experiment.

On average, the position and orientation discrepancies between the simulation and hardware experiments are 5 cm and 12°. Such differences can be deemed reasonable since we playback the planned trajectories on the robot using a naive joint-level controller, i.e., without a closed-loop controller that tracks and compensates for the deviation of the object’s trajectory from the planned one. The deviations of the executed motions from the planned motions are expected considering errors caused by modeling and perception. The main goal of this study is to show that the proposed method can solve for complex tasks by exploiting the smooth virtual forces and the residual non-physical forces do not hinder the task performance.

## 5 *In-situ* Terrain Classification and Estimation

Robust locomotion on deformable terrains is necessary for biped humanoid robots to perform tasks effectively in unstructured environments. The knowledge of deformable terrain properties, particularly the stiffness, has major implications in modeling the robot walking dynamics, which is the key to achieve stable gait patterns. Prior studies on walking stabilization chose to model such walking dynamics using pre-identified stiffness or damping constants (Wittmann et al., 2016; Wu et al., 2018; Hashimoto et al., 2012). However, it is unlikely for robots to access such terrain properties in advance when deployed to unknown environments.

In this section, we present an *in-situ* ground classification and estimation method that can be used to improve the stability of the robot while traversing unknown terrain, utilizing NASA’s humanoid robot Valkyrie (Radford et al., 2015). The terrain estimation works in two steps: i) The robot tries to identify the terrain type from a database. If the terrain is recognized, all needed data can be retrieved and used by the controller. ii) If the terrain is classified as an unknown type, the robot then estimates its stiffness by using Bernstein-Goriatchkin (Ding et al., 2013; Caurin and Tschichold-Gurman, 1994) pressure-sinkage model. The estimated stiffness can then be fed to stabilizers such as the one proposed in (Hashimoto et al., 2012).

### 5.1 Related Work

Our study on robot foot-terrain interaction is inspired by (Skonieczny et al., 2014), where the interaction between soil and single wheel is analyzed using optical flow techniques. Computer vision techniques have been widely used for terrain classification in the past (Weiss et al., 2008; Brandão et al., 2016). However, due to the poor lighting conditions in the outer space, it is desirable to augment vision-based techniques with a terrain classification approach that relies on physical contacts between the robot foot and the terrain. We thus aim at providing a “sense-of-walking” to the robot by using on-board sensors. In (Walas et al., 2016; Otte et al., 2016), ankle mounted force/torque sensors and accelerometers are used, respectively, to achieve terrain classification. Our approach is comparable to (Otte et al., 2016) as Recurrent Neural Networks (RNNs) are used but they differ in the aspect that we perform terrain classification with a bipedal robot while (Otte et al., 2016) uses a wheeled mobile robot. To describe terrains’ properties under pressure, various pressure-sinkage models have been studied in the past (Komizunai et al., 2010; Ding et al., 2013). There is no common opinion on which model is better than others. We choose Bernstein-Goriatchkin model considering it is one of the most commonly used models and it is relatively easy to implement. The method we developed for terrain estimation can be viewed as an extension of (Will Bosworth1 and Hogan, 2016), where spring model is used to estimate the ground stiffness by measuring the force and leg displacement during the interaction between a quadrupedal robot, Super Mini Cheetah (SMC), and the ground. Since SMC has spherical shaped feet, point contacts are used when modeling the foot-terrain interaction. This is not applicable for bipedal robots with flat soles because the support force on sole is distributed unevenly. To overcome this challenge (Caurin and Tschichold-Gurman, 1994), develops a special sensor system consisting of multiple sensor cells to measure the force distribution. Instead of using additional sensors, our approach achieves estimation for stiffness using a single load cell which provides only a net force measurement.

### 5.2 Terrain Classification

**FIGURE 8:**
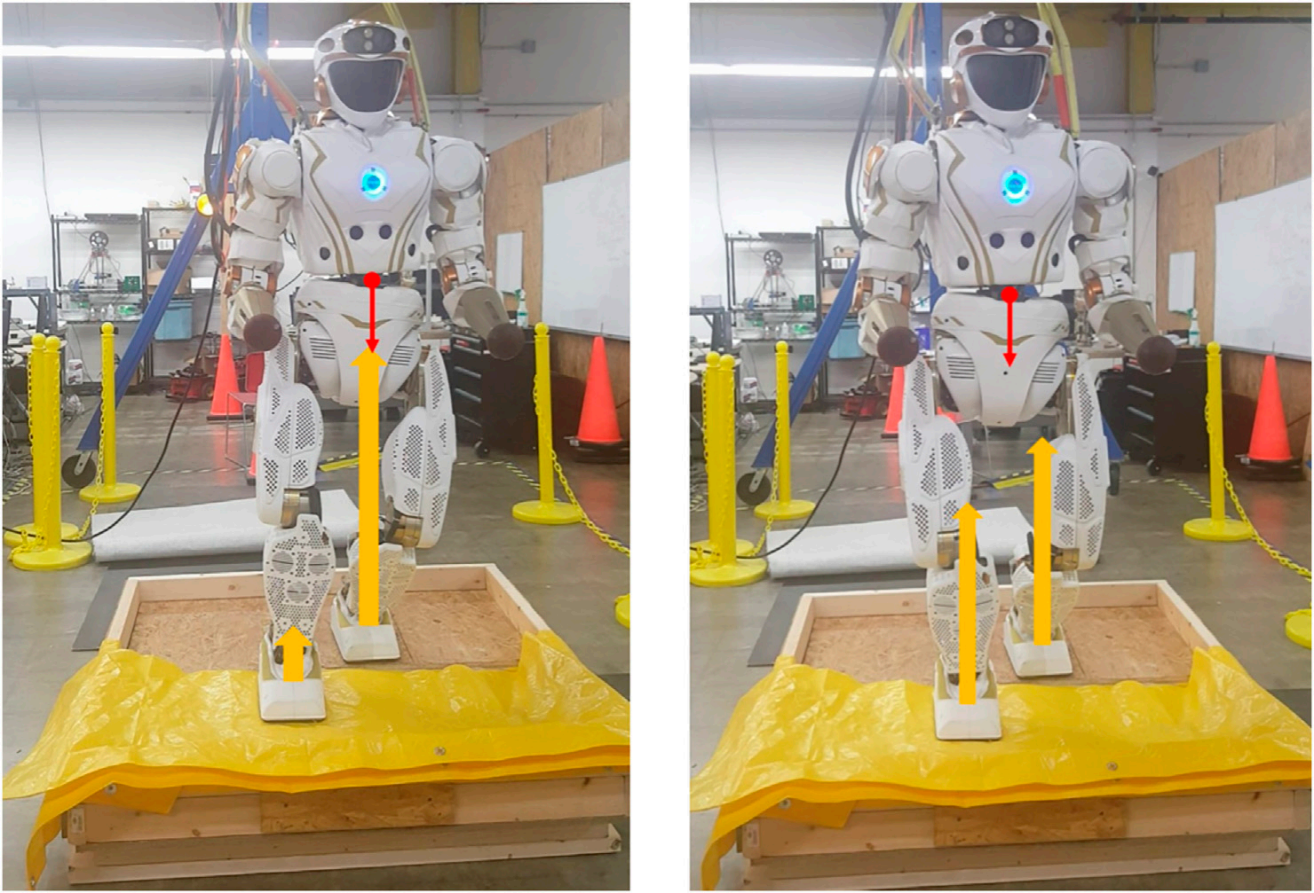
Valkyrie is performing the touch-land motion. The red arrow represents CoM and yellow arrow represents support force on each foot.

To study the foot-ground interaction, we designed a set of experiments involving Valkyrie interacting with four types of terrains, including wood mulch, rubber mulch, mason sand, and gym foam mats. A custom sandbox, as seen in Figure 8, was built for Valkyrie to stand in and perform a set of pre-designed motions. Half of the sandbox is covered by a wood plate, where Valkyrie can stand stably, and the other half is filled with specified deformable ground materials to be tested. The sandbox is not used when testing on the gym foam mat, which Valkyrie can stand on directly. The data for terrain classification and estimation is collected by commanding Valkyrie to perform a touch-land motion.

Valkyrie starts from standing on the wood plate of the sandbox and then moves the right foot onto the terrain. During this motion, Valkyrie slowly moves the center of mass (CoM) from the left foot to the center of the two feet (Figure 8). The support force on the right foot increases from zero to approximately 650 N, which is about half the weight of Valkyrie, in this process. Valkyrie then moves the right foot back onto the wood plate and the motion is finished. The right foot force and torque changes during this motion are measured by the load cell. Meanwhile, the right foot sinkage in the terrain is also calculated using joint positions.

One method to calculate the foot sinkage, *δ*, is to use the kinematic chain of the robot. Transformation matrix from coordinate frame *RightFoot1* to *RightFoot2* is denoted with TR1R2 representing the transformation of the right foot at two different poses. *RightFoot1* corresponds to the foot pose when the foot makes contact with the terrain. Since Valkyrie keeps its foot flat during the swing, it is reasonable to take *RightFoot1* as the fixed ground frame. *RightFoot2* is the frame when the foot sank in the soft terrain. There could be many different choices for *RightFoot2*. Using the transformation TR1R2, we can attain the position for each specific point of the sole relative to ground as TR1R2⋅P, where $P={\left[x,y,0,1\right]}^{T}$ is the coordinates in *RightFoot2* frame. The sinkage $\delta_{xy}$ can then be represented as the third element of TR1R2⋅P, denoted as TR1R2⋅P⋅P(3). To get TR1R2, we introduce a third coordinate frame *LeftFoot*. Since Valkyrie’s left foot is stationary during the experiments (right foot taking the step), we take *LeftFoot* as the reference coordinate frame. Using the robot’s kinematic chain, we can get TLR1, TLR2, and thus TR1R2.

We use RNNs, particularly Long Short-Term Memory (LSTM) network, to classify the terrain. Comparing to other classification methods such as Support Vector Machine (SVM), the use of RNNs gains us two advantages. First, our method can be applied to a raw data stream rather than accumulated data over time, thus, we can achieve terrain classification in real-time. Second, it has been shown that RNNs have better performance than SVM (Otte et al., 2016) and we achieved 95% accuracy on average during experiments.

Fifteen features are selected as input for the LSTM network, which are: right foot force in *X, Y, Z* directions, right foot torque in *X, Y, Z* directions, pitch/roll angular displacement/acceleration of the right ankle, pitch/roll angular torque of the right ankle, and the right foot sinkage, represented by the first three elements on third row of the transformation TR1R2. One data set is collected when Valkyrie performs one touch-land motion for one specific terrain. In total, we collected 30 data sets for the foam mat, 30 data sets for the wood mulch, 34 data sets for the rubber mulch, and 42 data sets for the sand. 90% of the data sets (118 data sets) are used for training and the rest (18 data sets) are used for testing. The LSTM network thus has 15 input neurons, one hidden layer with 64 hidden blocks and one linear output layer with four neurons. A softmax function is used to determine the terrain type. For each experiment run, the model is trained with a fixed learning rate of 0.0005 for 16 epochs. To track the performance of our model, the prediction accuracy is calculated using the test data set after each training epoch. Out of the five experiment runs, we achieved 100% prediction accuracy for four runs and 89% accuracy for the remaining one.

### 5.3 Terrain Estimation

Although the RNN based classification method works well, there are chances that Valkyrie may encounter with terrains of unknown type. To handle such situations, a method that can estimate an unknown terrain’s stiffness, which governs the foot sinkage behavior under load, is desired.

#### 5.3.1 Terrain Stiffness Model

**FIGURE 9:**
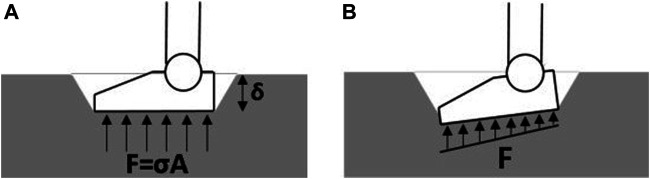
Contact between the foot and ground terrain. **(A)** Even sinkage when the contact is flat. **(B)** Non-even sinkage when the contact is oblique.

To describe the terrain’s stiffness, the well-known pressure-sinkage model developed by Bernstein-Goriatchkin (Caurin and Tschichold-Gurman, 1994; Ding et al., 2013) is used:

$$\sigma =k\cdot \delta^{n}$$ (35)

where σ(Pa) is the normal pressure, $k\left(Pa/m^{n}\right)$ and n(non−dimensional) are the terrain parameters and δ(m) is the foot sinkage in terrain. The two terrain parameters, *k* and *n*, are to be estimated by using data from Valkyrie’s on-board sensors. The challenge here is that Valkyrie uses only one 6-axis load cell mounted at the ankle to measure support force acting on the foot, which cannot provide a detailed force distribution upon sole. Ideally, Bernstein-Goriatchkin model can be easily implemented to determine the terrain stiffness when the terrain undergoes an even and flat sinkage, as seen in Figure 9A. However, when a bipedal robot walks on deformable terrains, the sinkage is usually non-uniform, thus the contact surface between the foot and the ground could be oblique, as seen in Figure 9B.

In this case, it is impractical to identify the sinkage value corresponding to the pressure calculated from the measured force on ankle. To handle this mismatch, we calculate the net force acting on the sole resulting from different levels of sinkage using the following equation

$$F=\int_{A}\sigma \left(x,y\right)\;dA=\overset{y_{2}}{\underset{y_{1}}{\int }}\overset{x_{2}}{\underset{x_{1}}{\int }}k\cdot \delta_{xy}^{n}\;dxdy$$ (36)

where *F* is the net force acting on the foot, $\delta_{xy}$ is the local foot sinkage at (x,y) expressed in the foot frame. The contact area between the foot and the ground is defined by (x1,x2) and (y1,y2), which are also expressed in the foot frame. These same parameters are used to calculate the torque values for verification, thus we pick the projection of the ankle in the foot frame as the origin. The contact area for Valkyrie is then defined as (−0.09 m, 0.18 m) along the *x*-axis and (-0.08 m, 0.08 m) along the *y*-axis based on the physical dimensions of the robot. Since *F* can be measured directly, using Eq. 36, we obtain *k* and *n* once we know the sinkage at different positions of the foot, i.e., $\delta_{xy}$. The sinkage $\delta_{xy}$ can be calculated using the same frame transformation method described in Section 3. Eq. 36 can then be written as

F=∫y1y2∫x1x2k⋅(TF1F2⋅P(3))ndxdy (37)

Theoretically, if two sets of *F* and TF1F2 for the sinking foot of different states are provided, we can solve for *k* and *n* using Eq. 37. It is worth mentioning that in Eq. 37, both transformation TF1F2 and support force *F* can be calculated or measured in real-time. Contact range *x* and *y* are pre-defined based on the foot dimensions. Therefore, this method can estimate the unknown terrain’s stiffness *in-situ*.

#### 5.3.2 Experiments and Results

**FIGURE 10:**
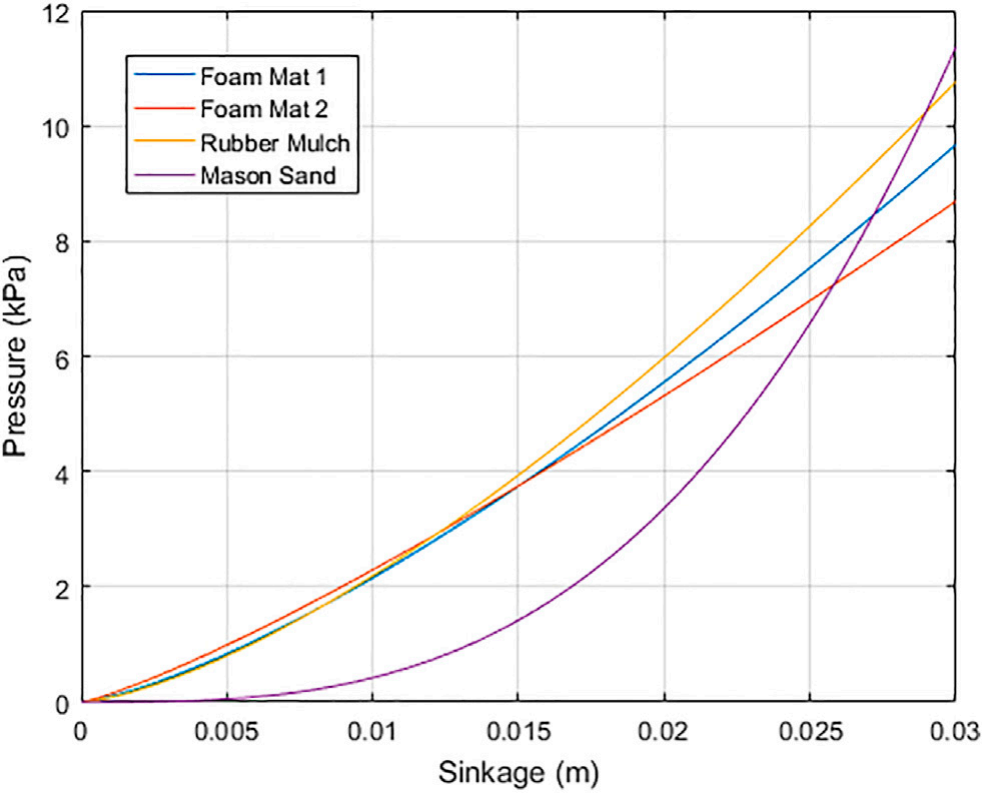
Relationship of pressure and sinkage for different terrains using estimated parameters.

To test the proposed estimation method, we run four experiments: two with gym foam mats (Gym Foam Mat one and two), Rubber Mulch, and Mason Sand. The same touch-land motion as described in Section 5.2 is performed by Valkyrie to collect data for terrain estimation. The support force acting on Valkyrie’s right foot and the frame transformation TF1F2 are recorded during each touch-land motion. Nonlinear Least-Squares Data Fitting (Coleman and Li, 1996) is then used to solve for parameters *k* and *n*. Table 2 shows the estimated parameters for four tested terrains. The pressure and sinkage relationships of the four terrains with the estimated parameters are depicted in Figure 10. We intuitively realize that the estimation results are not valid for the sand. This is because the sand is known to be stiffer than both foam and rubber mulch but Figure 10 shows that the sand is estimated to be the softest. The reason of inaccurate estimation for sand is that the sand demonstrates a much smaller deformation under the same pressure compared with the form and the rubber mulch. During the experiments, the foam mat and the rubber mulch have a deformation between 20 and 25 mm while the sand has a deformation between 5 and 10 mm, which is a section that is too short to do regression for solving *k* and *n*.

**TABLE 2:** Estimated stiffness parameters *k* and *n* for tested terrains using Bernstein-Goriatchkin Model.

Terrain	k ($10^{3}Pa/m^{n}$)	N	Validity
Gym foam mat 1	1167.861	1.367	Good
Gym foam mat 2	609.296	1.212	Good
Rubber mulch	1738.93	1.45	Good
Mason sand	421146.385	3.00	Poor

To validate the estimation results for the foam mats and the rubber mulch, we compare ankle torques measured from the load cell with ankle torques calculated using the estimated parameters. We choose torque comparison as the validation approach for one practical reason: many bipedal robots walking algorithms keep the robot balanced by tuning torques at the ankle (Kim et al., 2007; Kang et al., 2012). We thus posit that a set of terrain parameters that can correctly predict torque changes can be useful in walking algorithms for keeping robots stable on deformable terrain. When Valkyrie’s foot lands on the terrain, the torque measured at the ankle should be equal to the torque caused by the force acting on the sole around the ankle axis. Considering the torque caused by gravity, we have.

$$\tau_{ankle}=\tau_{f}+\tau_{g}$$ (38)

where $\tau_{ankle}$ is the net torque measured at the ankle; $\tau_{g}$ is the torque due to gravity, which equals to the measured torque at the ankle during the swing; $\tau_{f}$ is the torque due to support force resulting from terrain deformation. Since we now have parameter *k* and *n* for the terrain, using Eq. 36, the pitch and roll torque can then be calculated as

$$\tau_{pitch}=\overset{y_{2}}{\underset{y_{1}}{\int }}\overset{x_{2}}{\underset{x_{1}}{\int }}k\delta_{xy}^{n}x\;dxdy,\;\tau_{roll}=\overset{y_{2}}{\underset{y_{1}}{\int }}\overset{x_{2}}{\underset{x_{1}}{\int }}k\delta_{xy}^{n}y\;dxdy$$ (39)

**FIGURE 11:**
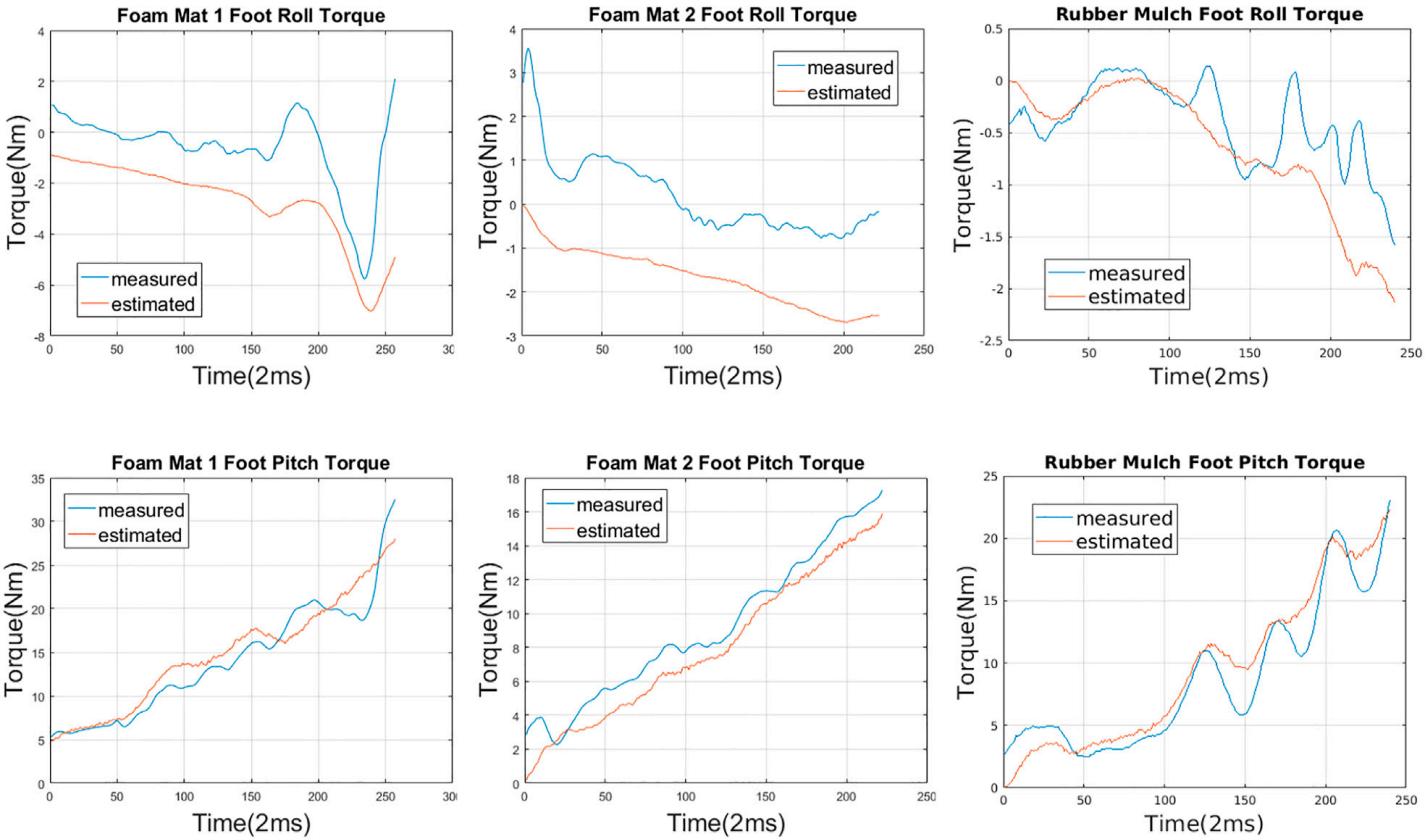
Foot pitch and roll torque comparison. Blue curves stand for measured torques and red curves represent the estimated torques.

Figure 11 shows the comparison between the calculated foot pitch and roll torques using estimated parameters and measured foot pitch and roll torques from the ankle load cell. The blue curves represent the measured results while the red curves stand for the estimated results. The plots show that the estimated parameters can predict the foot pitch torque well. As for the foot roll torque, the estimated results match the trend of torque changes with an offset of 1.5 Nm on average. One possible source of this offset is the friction between the foot and the terrain. The friction coefficient between the foot and the terrain is currently not estimated and is planned as the future work.

To numerically examine how well the estimated parameters predict the foot pitch torque, we calculate the Normalized Root-Mean-Square Error (NRMSE) between the estimated pitch torques and the measured pitch torques. The Gym Foam Mat one and two show a NRMSE of 15.9 and 15.7%, respectively, which is the equivalent to an accuracy of 84.1 and 84.3%. While the Rubber Mulch showed an NRMSE of 24.2%, which is an accuracy of 75.8%. Since the estimated parameters from the Gym Foam Mat one and two and Rubber Mulch experiments can well predict foot pitch torque (an average accuracy of 80%) and roll torque (same trend but with an offset), we assert that the proposed method can be used to measure the stiffness of an unknown deformable terrain. Future work will focus on improving the estimation accuracy, including estimation of friction coefficient for a thorough knowledge of unknown terrains.

## 6 User Interface

Supervisory-control interfaces are an important aspect to robots in extreme environments as you often want a human-in-the-loop to help evaluate and make decisions for critical objectives of a task at hand. In any supervisory-control interface, it is important to find the balance of control between robot and human. Too much control or autonomy on the robot side leaves the human unable to potentially provide valuable feedback to the robot. While too much control on the human side can leave the operator overwhelmed with information and decision making, leaving less focus for the critical elements of a given task. Additionally, too much control on the human side tends to increase the interface complexity and therefore increase the amount of required operator training to utilize the interface.

In this section, we present our work on a computer-based, supervisory-control interface for NASA’s humanoid robot Valkyrie (Radford et al., 2015), that aims to balance the supervisory-control to allow for relatively untrained operators. Our interface was originally designed to accomplish the tasks put forward by the Space Robotics Challenge, a challenge using a simulated Valkyrie robot in a virtual, mock-up Mars environment with tasks such as turning valves, moving equipment, and inspection. However, our interface was purposely designed to be generic enough to accomplish a wide variety of tasks and not require tasks to be pre-programmed into the interface and is therefore extendable to a wide variety of domains.

### 6.1 Related Work

The DARPA Robotics Challenge (DRC) was a two-year challenge with the goal of accelerating progress in human-supervised robotic technology for disaster-response operations. The DRC helped support development for a variety of robotic research, with one key area being the design of human-robot interaction with focus on supervisory control methodologies (Yanco et al., 2015; Norton et al., 2017). The DRC allowed for a variety of supervisory-control interfaces for humanoid robots to be developed furthering the state-of-the-art. However, most of the developed interfaces required multiple well-trained operators in order to successfully complete tasks (Norton et al., 2017). Several interfaces aimed to reduce the amount of input required on the human operator, but still allowed operators to take over low-level commands of the robot when needed and provided a wide range of robot sensor data (Stentz et al., 2015; DeDonato et al., 2017).

### 6.2 Interface

Our Valkyrie interface was designed to mimic several of the humanoid interfaces designed for the DRC finals, but with a reduced number of capabilities, inputs, and sensor data to allow for both a single operator and to reduce operator training time. The goal was to create a comparable interface to ones used at the DRC, but user-friendly enough to allow for non-experts to quickly learn and operate.

**FIGURE 12:**
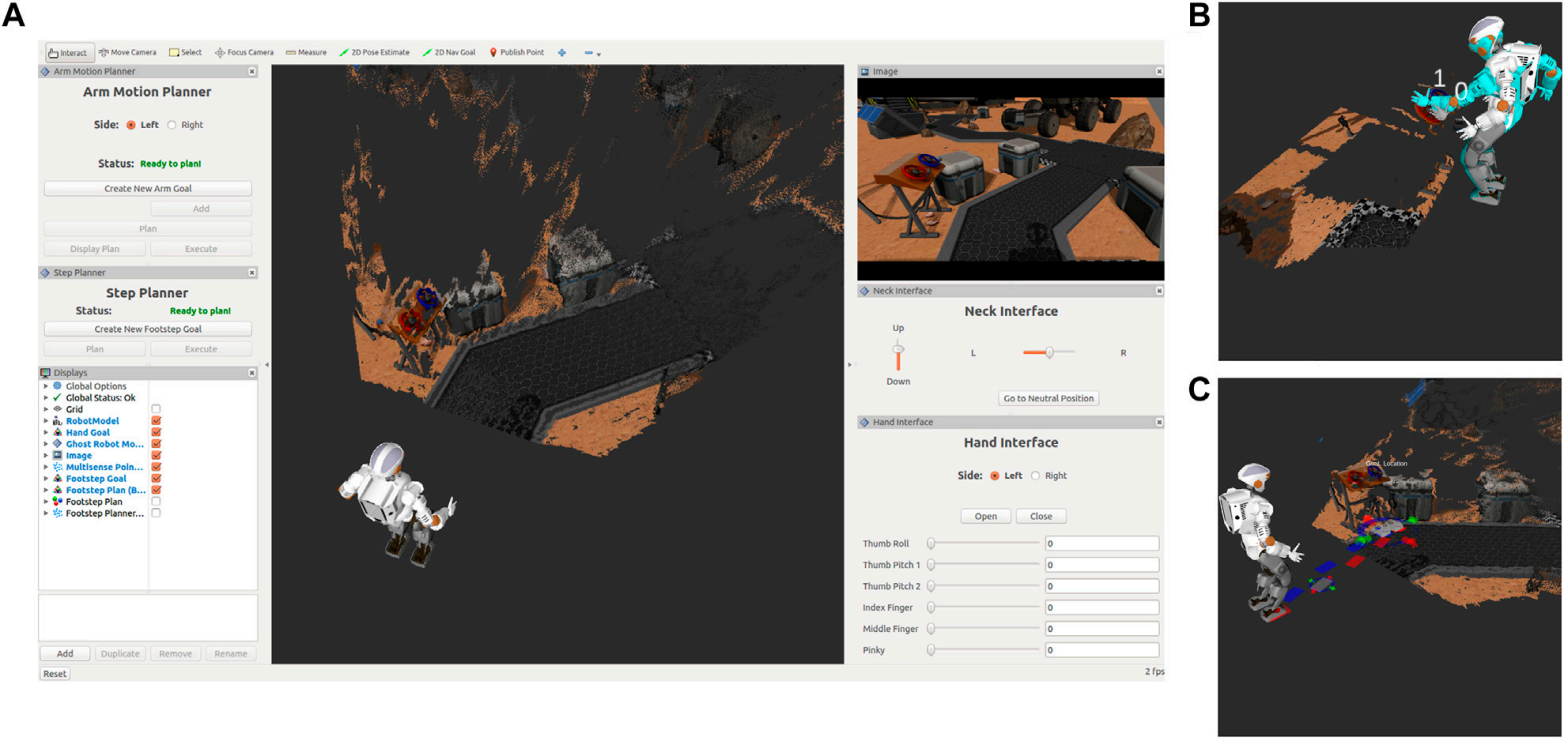
Valkyrie interface.

Our interface, as seen in Figure 12, utilizes RViz, a 3D visualization tool for ROS, and custom Qt Widgets, also known as Panels in RViz. RViz was chosen as the interface backbone since ROS is widely used in the robotics community making it familiar to many and for its built-in data visualization tools. RViz was also used for several of the interfaces designed for DRC (DeDonato et al., 2015; Kohlbrecher et al., 2015; Stentz et al., 2015). The interface can be broken down into four custom panels: Arm Motion Planner, Step Planner, Neck Interface, and Hand Interface. Additionally, there are three built-in RViz panels: the main visualization window, a display panel, and a camera feed panel.

The main window consists of the built in 3D data visualization of RViz and for our specific interface includes a point-cloud from Valkyrie’s Multisense SL sensor located in the head, a robot model of Valkyrie’s current joint state and planned joint state from the arm motion planner, and interactive markers to interface with the arm motion and step planners. The display panel allows users to select what is actively visualized in the main window and the camera feed panel for our interface is a camera feed from one of the stereo cameras in the Multisense SL.

#### 6.2.1 Arm Motion Planner Panel

The Arm Motion Planner panel utilizes a whole body inverse kinematics solver that finds motion plans based on a set of cost and constraints (Long et al., 2016, 2017). Cost and constraints can be categorized as either kinematic, collision avoidance, or ZMP. To reduce the complexity of the interface, operators only need to define the desired end-effector positions and a predefined set of cost and constraints are used, i.e., balancing constraint or velocity cost, etc. To define the end-effector positions, operators can add waypoints for the end-effector to traverse though. This is accomplished through the use of interactive markers that represent a waypoint and are visually represented as a single end-effector. Operators are able to translate and rotate the waypoint by using arrows and circular scroll bars that surround the marker visual. The waypoints are ordered to allow for a variety of paths. A plan can be requested after the waypoints have been placed in a satisfactory manner. Operators are provided a planning indicator and are informed when the planning is complete and whether the planner was successful in finding a plan or unsuccessful. If a plan returns unsuccessful, then the returned plan either violated one of the default set of costs or it was unable to traverse through all the placed waypoints. Operators are notified of the reason the planned returned unsuccessful and informed on how to improve the waypoints to results in a successful plan. For example, a waypoint may be out of the robot’s workspace and need to be moved closer to the robot. After a plan is returned, the operator can visualize the robot’s movement through the entire plan before allowing the robot to execute it to ensure that the plan both provides the desired outcome and is collision free. Figure 12B demonstrates an example of placed waypoints and the final robot configuration of the returned plan. After visualizing the plan, operators can request the robot execute it.

#### 6.2.2 Step Planner Panel

The Step Planner panel allows operators to designate a goal position for the robot to navigate to. It works in a similar strategy as the Arm Motion Planner panel, except rather than multiple waypoints, there is only a single waypoint that represents the final goal pose for feet with a visual of a pair of feet. Additionally, the interactive marker that represents the waypoint only allows for 2D interaction on a plane. After this single waypoint is placed, operators can request a plan which is generated using an A* search-based footstep planner. Same as the Arm Motion Planner panel, operators are provided a planning indicator and informed when the plan returns. As soon as a successful plan is found the planner will return. However, the planner will infinitely plan until a successful plan is found, therefore, there is a 10 s timeout in place to force a return and the operator is notified of this timeout and to try again. As soon as a successful plan is returned, colored interactive markers that represent each footstep in the returned plan are visualized, red for the right foot and blue for the left foot. These interactive markers can be adjusted by the operator by clicking on the footstep marker to activate the interactive marker controls and then adjusting the footstep in the same method as the waypoints, the interactive marker controls can also be switched off by clicking the footstep marker. Figure 12C shows an example of a placed goal marker, a returned footstep plan, and selected footstep in adjustment mode. When finished making adjustments, operators are then able to send the plan to the robot for execution.

#### 6.2.3 Neck Interface and Hand Interface Panels

The Neck Interface and Hand Interface panels both work in the same manner with a series of joint sliders and buttons with some predefined joint positions. The Neck Interface panel allows operators to move the head of the robot right-left and up-down with sliders and a predefined neutral position, where the robot is looking straight forward. The Hand Interface panel has sliders for each individual finger to open-close along with two predefined open hand and closed fist grasp. Sliders were added over additional grasp types to allow the operator to position the hand at any intermediate pose if needed.

## 7 Conclusion

Humanoid robots are equipped with the necessary combination of location and manipulation capacities to fulfill a variety of roles in man-made hazardous environments. However, there are numerous challenges before this potential can be realized. In this paper, we presented our research to help further the development of utilizing humanoid robots in supervisory roles for extreme environments, with emphasis on operation in nuclear facilities.

We first examined ways to improve manipulation within constrained environments, with a focus on glovebox operation, by looking into three different areas. First, we aimed to better understand a robot’s feasible workspace in constrained environments by developing a method called the constrained manipulability polytope (CMP) that considers both the robot’s capabilities and the constraints imposed by an environment. The CMP methodology assists in both autonomous motion planning and human-in-the-loop teleoperation by providing feedback of the robot’s workspace to the operator and indeed by providing a representation of both robot joint and Cartesian space constraints as a bounded limits in tool motion. Second, we investigated using contact supports to increase stability and we presented our theoretical methodology to accomplish contact-implicit motion planning. The objective of this work was to increase the robot’s workspace by using support contacts to reach areas that are not within the robot’s workspace without support. Third, we considered using non-prehensile manipulation over dexterous manipulation and presented a methodology capable of pushing objects in a constrained environment. The purpose of this work was to add the capability of moving objects around that might not be within reach of the robot or easily manipulated with dexterous hands.

We also detailed methods to improve locomotion for humanoid robots by developing a technique to accomplish *in-situ* terrain classification and estimation that can be used to better inform a walking controller. Finally, we presented our generic humanoid robot interface that allows for robot operation from operators with limited training and/or knowledge of robotics. When operating in high-risk environments, such as nuclear facilities, it will be necessary to keep humans-in-the-loop especially individuals with expertise in the environment the robot is operating in. Future work should consider further developing all these areas and combining them into one robotic platform with a supervisory-control interface for a single operator. Human supervised robots will be a key role in the future operation, maintenance, and decommissioning of nuclear facilities.

## Data Availability

The original contributions presented in the study are included in the article/Supplementary Material, further inquiries can be directed to the corresponding author.

## Footnotes

1 We use the C++ wrapper for Fukuda’s cdd library available here: https://github.com/vsamy/eigen-cdd, while the MATLAB^©^ computations used the Multi-Parametric Toolbox 3.0 (Herceg et al., 2013).

2 Information regarding the glovebox (https://www.belart.com/bel-art-h50026-0000-sidentry-glove-box-30-x-24-x-24.html): and the objects (http://www.melissaanddoug.com/deluxe-jumbo-cardboard-blocks---40-pieces/2784.html): are available online.
